# Isolation, characterization, and pathogenicity assessment of *Corynebacterium pseudotuberculosis* biovar equi strains from alpacas (*Vicugna pacos*) in China

**DOI:** 10.3389/fmicb.2023.1206187

**Published:** 2023-07-03

**Authors:** Wanyu Meng, Shanyu Chen, Lin Huang, Jinpeng Yang, Wenqing Zhang, Zhijun Zhong, Ziyao Zhou, Haifeng Liu, Hualin Fu, Tingmei He, Guangneng Peng

**Affiliations:** ^1^The Key Laboratory of Animal Disease and Human Health of Sichuan Province, College of Veterinary Medicine, Sichuan Agricultural University, Chengdu, Sichuan, China; ^2^Sichuan Wolong National Natural Reserve Administration, Wenchuan, Sichuan, China

**Keywords:** alpaca, *Corynebacterium pseudotuberculosis*, biovar equi, virulence, antibiotic resistance, pathogenicity, genomic sequencing

## Abstract

*Corynebacterium pseudotuberculosis* is a zoonotic pathogen that causes lymphadenitis in humans, livestock, and wildlife. In this study, *C. pseudotuberculosis* biovar equi strains were isolated from three alpacas. Antibiotic susceptibility tests and pathogenicity tests were also conducted. Moreover, one strain was sequenced using DNBSEQ and Oxford Nanopore technology. The three strains exhibited resistance to aztreonam, fosfomycin, and nitrofurantoin. The median lethal doses (LD_50_) of strains G1, S2 and BA3 in experimentally infected mice was 1.66 × 10^5^ CFU, 3.78 × 10^5^ CFU and 3.78 × 10^5^ CFU, respectively. The sequencing of strain G1 resulted in the assembly of a chromosomal scaffold comprising 2,379,166 bp with a G + C content of 52.06%. Genome analysis of strain G1 revealed the presence of 48 virulence genes and 5 antibiotic resistance genes (ARGs). Comparative genomic analysis demonstrates a high degree of genetic similarity among *C. pseudotuberculosis* strains, in contrast to other *Corynebacterium* species, with a clear delineation between strains belonging to the two biovars (ovis and equi). The data of the present study contribute to a better understanding of the properties of *C. pseudotuberculosis* biovar equi strains and the potential risk they pose to alpacas and other livestock, as well as the necessity of ongoing surveillance and monitoring of infectious diseases in animals.

## 1. Introduction

*Corynebacterium pseudotuberculosis* is a facultative intracellular pathogen, which belongs to the *Corynebacterium*, *Mycobacterium*, *Nocardia*, and *Rhodococcus* (CMNR) group of the *Actinomycetota* (Mills et al., 1997; Dorella et al., 2006). It is a non-motile, non-capsulated, and non-sporulating Gram-positive bacterium, with fimbriae (Hard, 1969). This zoonotic pathogen is the main cause of caseous lymphadenitis (CLA) and can infect humans (Peel et al., 1997), livestock (Oliveira et al., 2014), and wildlife (Oliveira et al., 2016) worldwide through respiratory, digestive and wound transmission. *C. pseudotuberculosis* causes (Kinne, 2016) serious economic losses, including reduced reproductive efficiency and losses in wool, meat, and milk production, as well as increased expenses for the drugs and labor required for treatment (de Sá Guimarães et al., 2011). It is classified by host preference and nitrate reduction into two biovars: ovis (serotype I, nitrate-negative) causes lymphadenitis in small ruminants, mainly sheep and goats; equi (serotype II, nitrate-positive) results in ulcerative lymphangitis in equines (Bernardes et al., 2021) and oedematous skin disease (OSD) in buffaloes (Barakat et al., 1984; Moussa et al., 2016). However, *C. pseudotuberculosis* exhibits phenotypic intra-species variation, with certain strains isolated from sheep exhibiting nitrate reduction capability, and some equine isolates displaying nitrate negativity (Costa et al., 1998; Connor et al., 2000; Retamal et al., 2011). Considering the potential discrepancies due to mutations, genotypic tests have proven to be a more reliable and sensitive diagnostic tool compared to phenotypic tests (Asfour and Darwish, 2014; Almeida et al., 2017) The *narG* (nitrate reductase gene) gene was also included in PCR assays along with the 16S rRNA, *pld* (Phospholipase D) and *rpoB* (RNA polymerase beta subunit region) genes to improve the accuracy of multiplex PCR at the biovar level (Almeida et al., 2017).

CLA is one of the most serious infectious diseases in alpacas and has been reported in Germany (Sting et al., 2022), USA (Anderson et al., 2004), and Peru (Braga et al., 2006). In China, case of *C. pseudotuberculosis* infection have been documented in sheep (Liu, 2021), goats (Wang R. et al., 2021), Bactrian camels (Liu et al., 1986; Guo et al., 1988), buffaloes (Zhang et al., 1989), chickens (He et al., 1991), deer (Zhu and Lei, 2001), and humans (Du, 1997). Among these, the strains isolated from goats were identified as biovar ovis through phenotypic and genotypic tests, while strains isolated from other hosts were characterized only phenotypically, without fully determining their biovars. Strains isolated from chickens and deer were nitrate-negative, whereas strains isolated from humans were nitrate-positive. Both nitrate-positive and nitrate-negative strains have been reported in Bactrian camels. However, to the best of our knowledge, there have been no documented cases of CLA in alpacas in China.

The pathogenic mechanism of *C. pseudotuberculosis* is closely associated with its virulence factors, including several virulence genes, such as *pld*, iron uptake and regulation (*fagA*, *fagB*, *fagC*, *fagD*), mycolic acid, σ factor, and oligopeptide permease (*oppA*, *oppB*, *oppC*, *oppD*, *oppF*) (Billington et al., 2002). PLD is a highly potent exotoxin secreted by *C. pseudotuberculosis*, known for its necrotic and hemolytic activities (Sutherland et al., 1989). It is widely regarded as the most critical virulence factor produced by the bacterium, and the *pld* gene has been found to be widely detected in all *C. pseudotuberculosis* strains isolated from mammals (Songer, 1997; Aquino De Sá et al., 2013). Selective extracytoplasmic function (ECF) sigma factor family proteins, such as *sigE*, mainly regulate cell surface stress and control the expression of virulence-associated genes in various pathogenic bacteria (Kazmierczak et al., 2005). The Opp transporters are not only essential for nutrient accumulation but also contribute to the regulation of diverse intercellular signaling processes. One notable aspect of their regulatory function is the control they exert over the expression of virulence genes in pathogenic bacteria (Samen et al., 2004).

With the continuous advancement of sequencing technology, the number of available whole genome sequences for *C. pseudotuberculosis* has been growing. At present, a total of 125 *C. pseudotuberculosis* strains from 19 countries and regions have been fully sequenced (as of April 1, 2023). This abundance of genomic data provides a valuable resource for conducting comprehensive comparative genomics studies. Of these, only one isolate was yielded from camel (biovar ovis) and one isolate from llama (biovar equi), while the majority of *C. pseudotuberculosis* isolates were from goats (28%), sheep (24%), and horses (22%). Notably, the whole genome sequencing of *C. pseudotuberculosis* strain isolated from alpacas has not yet been reported (Data source: https://www.ncbi.nlm.nih.gov/genome/. The whole genome sequencing statistics of *C. pseudotuberculosis* are presented in Supplementary Table S1 and Supplementary Figure S1). All strains that infect sheep and goats are biovar ovis (strain PA02 was mistakenly labeled as biovar equi and is actually lacking the *narG* gene, as shown in Supplementary Figure S2). In contrast, all strains that infect horses and buffalo are biovar equi, while strains that infect cattle include both biovars ovis and equi. Seven *C. pseudotuberculosis* biovar equi strains that were isolated from equine were subjected to pan-genomic analysis, which revealed one of the smallest core genomes ever recorded and considerable genetic variability. There were no discernible genetic distinctions among the strains responsible for the three distinct types of infection, including those causing external abscesses, infections with abscess formation in the internal organs, and ulcerative lymphangitis (Barauna et al., 2017). The clustered regularly interspaced short palindromic repeats (CRISPR)-Cas (CRISPR-associated proteins) cluster is only seen in the biovar equi strains, while the methylation type III cluster is only found in biovar ovis strains (Parise et al., 2018).

The main objective of this study was to identify the causative bacteria responsible for the disease in three alpacas and investigate their biological characteristics, with a focus on pathogenicity assessed through mouse infection tests. Furthermore, it entailed the whole genome sequencing of the *C. pseudotuberculosis* biovar equi strain isolated from an alpaca, and this genome was compared to other *C. pseudotuberculosis* strains of both biovars ovis and equi.

## 2. Materials and methods

### 2.1. Sample collection

In November 2021, two diseased alpacas (G1 and S2) and a dead alpaca (BA3) from a local alpaca farm were brought to the Animal Hospital of Sichuan Agricultural University (Chengdu, Sichuan, China) for consultation. Tests for *Brucella* antibodies were negative for alpacas G1 and S2. Despite receiving careful medical attention, alpaca G1’s condition deteriorated, and it died on the third day of treatment. Necropsies were performed on the dead alpacas, and extremity pus from one diseased alpaca (S2) and liver abscesses from two dead alpacas (G1 and BA3) were aseptically collected.

### 2.2. Bacteriological examination

Samples were subjected to routine bacteriological examination using Luria-Bertani (LB) broth (Hopebio, Qingdao, China), including cultivation under both aerobic and anaerobic conditions. Three mono-colony isolates from one sick alpaca and two dead alpacas were marked as S2, G1, and BA3, respectively. The hemolytic ability of the strains was determined by inoculating them onto blood agar plates (Solarbio, Beijing, China) containing 5% sheep blood. Morphological and biochemical properties of the bacteria were characterized through gram staining and standard biochemical tests, according to the manufacturer’s instructions (Hopebio). The resulting isolates were stored at −80°C in 25% glycerol for subsequent experiments.

### 2.3. Molecular identification

The genomic DNA from the isolates was extracted using the Bacterial DNA Kit (Tiangen, Beijing, China) and stored at −80°C. For bacterial species identification, the partial 16S rRNA gene was amplified using the primers 27-F (5’-GATGGTCATAGGGATGAAGAGCTT-3′) and 1492-R (5’-AGGGATGAAGAGCTTCGGCTCTG-3′) (Fredriksson et al., 2013). Additionally, the *narG* gene was amplified using the primers F (5′-ACCCGTACTTGCACTCTTTC-3′) and R (5′-AGTCAGTACTTCCGCAGGTC′) (Almeida et al., 2017). Each PCR reaction (25 μL) contained 12.5 μL 2 × *Pro Taq* Master Mix (Accurate Biology, Hunan, China), 8.5 μL RNase-free H_2_O (Sangon Biotech, Shanghai, China), 1 μL forward and reverse primers (Sangon Biotech), and 2 μL template DNA. In the PCR test for amplifying the partial 16S rRNA gene, RNase-free water was used instead of template DNA as a negative control. For other PCR tests, *Staphylococcus aureus* ATCC 25923 was used as a negative control strain. The reaction conditions included predenaturation at 94°C for 5 min, followed by 35 cycles of 94°C for 1 min, annealing temperature for 50 s, and 72°C for 1 min, with a final extension at 72°C for 10 min. The PCR products were validated by 1% agarose gel electrophoresis with TS-GelRed (Tsingke Biotechnology, Beijing, China) and sent to Tsingke Biotechnology Co., Ltd. for Sanger sequencing. A homology search was performed in the GenBank database using the BLAST tool,^1^ and Clustal X2.0^2^ for sequence alignment.

### 2.4. Antibiotic susceptibility testing

The Kirby-Bauer disk diffusion method (Oxoid, Basingstoke, UK) was utilized to test the antimicrobial susceptibility of the isolates. Representative drugs from 12 major classes of antibiotics, including β-lactams, carbapenems, polyphosphates, glycopeptides, aminoglycosides, tetracyclines, macrolides, amide alcohols, quinolones, sulfonamides, nitrofurans, and rifampicin, were selected. The bacterial solution cultured to 0.5 McFarland turbidity was evenly spread on Muller-Hinton (MH) solid medium (Hopebio) using a spreader stick. Disks were then placed on the medium and the culture was incubated at 37°C for 24–48 h. *S. aureus* ATCC 25923 was employed as the quality control strain in our study. The zone diameters for each drug were interpreted based on the Clinical and Laboratory Standards Institute (CLSI, 2019) and recorded as sensitive (S), intermediate (I), or resistant (R).

### 2.5. Pathogenicity test

The animal study was reviewed and approved by the Sichuan Agricultural University Animal Ethical and Welfare Committee. Female Specific Pathogen Free (SPF) Kunming mice were obtained from Chengdu Dossy Experimental Animals CO., LTD. (Chengdu, China). The mice were housed in clean cages at 23 ± 2°C and a relative humidity of 50 ± 10%. The mice were 6 weeks old at the start of the study.

Preliminary experiments were conducted to determine the infection range of three *C. pseudotuberculosis* strains (6 × 10^4^ to 10^7^ CFU/mL) (CFU: Colony Forming Unit). Healthy mice (*n* = 78) were randomly divided into 13 groups of 6 mice each. Groups 1–4, 5–8, and 9–12 were intraperitoneally injected with 0.2 mL of G1, S2, and BA3 bacterial solutions (with normal saline and diluted to 6 × 10^4^ to 10^7^ CFU/mL), respectively. Group 13 served as the control group and received 0.2 mL of saline intraperitoneally.

Clinical signs and mortality were recorded every 2 h within 12 h post-injection and every day for 7 consecutive days. The median lethal dose (LD_50_) was calculated. Histopathological analysis was performed on the initial three mice that died following infection with strains G1, S2, and BA3, respectively. The liver, heart, spleen, lung, kidney, and intestines of dead mice were fixed in 10% formaldehyde for histopathological examination. On day 14, the organs of the remaining mice were collected and weighed to compare changes in organ index after infection with different concentrations of bacterial solutions.

### 2.6. Whole genome sequencing

#### 2.6.1. Genome sequencing and assembly

The genome of a *C. pseudotuberculosis* strain was sequenced using a combination of the DNBSEQ platform (BGI, Shenzhen, China) and the Oxford Nanopore technology (ONT, Oxford, UK) at the Beijing Genomics Institute. The program Canu was utilized for self-correction. Draft genomic unitigs were assembled using Canu, a high-quality assembler that employs a corrected circular consensus sequence subread set. To improve the accuracy of the genome sequences, GATK^3^ was used to make single-base corrections.

#### 2.6.2. Gene prediction and annotation

The functional annotation of the *C. pseudotuberculosis* strain genome for open reading frame (ORF) prediction was performed using glimmer3^4^ with Hidden Markov models. tRNA, rRNA and sRNA recognition made use of tRNAscan-SE,^5^ RNAmmer, and the Rfam database. The tandem repeat annotation was obtained using the Tandem Repeat Finder,^6^ and the minisatellite DNA and microsatellite DNA were selected based on the number and length of repeat units. The Genomic Island Suite of Tools (GIST) was used for genomic island analysis^7^ with IslandPath DIOMB, SIGI-HMM, IslandPicker method. Prophage regions were predicted using the PHAge Search Tool (PHAST) web server^8^ and CRISPR-Cas identification using the CRISPRCasFinder.^9^

The best hit was extracted using the BLAST alignment tool for function annotation. Virulence factors and antibiotic resistance genes (ARGs) were identified based on the core datasets in the VFDB (Virulence Factors of Pathogenic Bacteria) and ARDB (Antibiotic Resistance Genes Database) databases. Additionally, general function annotation was performed using three databases: COG (Clusters of Orthologous Groups), GO (Gene Ontology), and KEGG (Kyoto Encyclopedia of Genes and Genomes).

#### 2.6.3. Comparative genomics

(i) Structural Variation (Synteny). The genome sequence of the *C. pseudotuberculosis* strain was compared with that of the reference bacteria. (ii) FastANI (version 1.32) software was used to conduct the Average Nucleotide Identity (ANI) analysis. (iii) CD-HIT clustering analysis was conducted on the protein gene sets of the *C. pseudotuberculosis* strain investigated in this study and other *Corynebacterium* strains to obtain the core/specific/dispensable gene set. (iv) Gene families were constructed by pairwise aligning the protein sequences of multiple target genomes using the BLAST, eliminating redundancy with Solar (BGI), and clustering the alignment results using the Hcluster_sg software. (v) The phylogenetic tree was constructed by the TreeBeST^10^ using the method of neighbor-joining (NJ).

### 2.7. Statistical analysis

The *in vitro* experiment was performed in triplicate and the data were analyzed using SPSS software (IBM, Armonk, NY, USA). Statistical significance was determined by one-way ANOVA, and differences were considered significant at *p* < 0.05 and extremely significant at *p* < 0.01. The LD_50_ values were calculated by Probit analysis (Xiong et al., 2013). The difference in LD_50_ among the three *C. pseudotuberculosis* strains were examined by the Kruskal-Wallis test. All graphical presentations were generated by Adobe Illustrator 2020 (Adobe Inc., San Jose, CA, USA) and GraphPad Prism 9.0 (GraphPad Software, San Jose, CA, USA).

## 3. Results

### 3.1. Postmortem examination

All three alpacas exhibited purulent infections in the skin and subcutaneous tissue, with some abscesses that developed in “beaded” pattern. White, yellowish pus or caseous pus masses were observed after incision of the pustules. Additionally, skin ulceration and necrosis were observed (Supplementary Figure S3). Upon necropsy of dead alpaca (G1, BA3), multiple abscesses were found in subcutaneous tissue, anterior shoulder lymph nodes, and inguinal lymph nodes. Alpaca G1 was found to have abscesses in the liver and mesenteric lymph nodes, as well as hemorrhagic spots in the lung (Supplementary Figure S4).

### 3.2. Bacteriological examination

**Figure 1:**
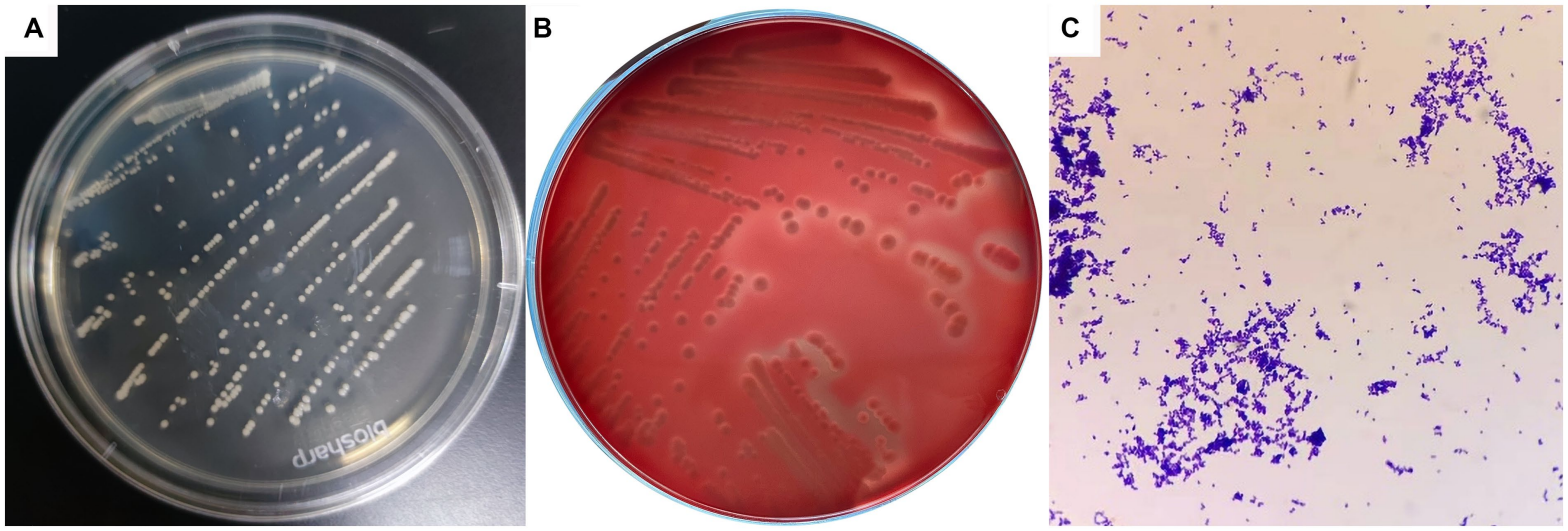
Colony morphology and bacterial morphology observation of *C. pseudotuberculosis*. **(A)** Colony morphology (LB solid medium). **(B)** Colony morphology (5% sheep blood). **(C)** Bacterial morphology (1000 ×).

Dry, friable, and opaque porcelain white colonies were grown on LB solid medium under both anaerobic and aerobic conditions (Figure 1A). A narrow and transparent hemolytic ring formed on the blood plate, which is β-hemolysis (Figure 1B). Microscopic examination of Gram-stained samples showed a large number of blue-purple globular or rod-shaped Gram-positive bacteria that were arranged singly and sporadically (Figure 1C).

Biochemical identification was consistent for the three strains (Table 1), and was in accordance with the biochemical properties of *C. pseudotuberculosis* previously summarized (Dorella et al., 2006). These three strains were further identified as *C. pseudotuberculosis* biovar equi by nitrate reduction experiments (+).

**Table 1:** Biochemical characteristics of *C. pseudotuberculosis* in this study.

Category	Result	Category	Result	Category	Result	Category	Result
Glucose	+	Sucrose	−	Hydrogen sulfide	−	Esculin hydrolysis	−
Mannose	+	Arabinose	−	Citrate	−	Urease test	+
Fructose	+	Xylose	−	Voges-Proskauertest	−	Catalase	+
Lactose	−	Raffinose	−	Gelatin liquefaction	−	Nitrate reduction	+
Maltose	−	Melibiose	−	Inositol	−		

+, positive; −, negative.

### 3.3. Molecular identification

Identification analysis of the partial 16S rRNA gene confirmed that all three isolates were *C. pseudotuberculosis* (accession numbers OQ980206, OP903368 and OP903369). These sequences exhibited 100% similarity to the sequences MT649220 (dromedary, equi, India) and LC549531 (goat, ovis, Malaysia), suggesting a high degree of similarity between the three strains.

The *narG* gene was successfully amplified and identified in all three isolates as *C. pseudotuberculosis* biovar equi (accession numbers OQ817706, OQ817707 and OQ817708). These sequences displayed 100% identity to both the sequences CP003652 (strain Cp162, equi, camel) and CP017384 (strain I37, equi, cattle).

### 3.4. Antibiotic susceptibility testing

The antibiotic susceptibility test showed that the three *C. pseudotuberculosis* strains were sensitive to all antibiotics tested except aztreonam, fosfomycin and nitrofurantoin. Specific results are shown in Table 2.

**Table 2:** Antibiotic susceptibility of *C. pseudotuberculosis* in this study.

Classification	Antibiotics	Judgment		
		G1	S2	BA3
β-lactams	Amoxicillin-clavulanate	S	S	S
	Ampicillin-sulbactam	S	S	S
	Cephazolin	S	S	S
	Aztreonam	R	R	R
	Penicillin	S	S	S
	Ceftriaxone	S	S	S
Carbapenems	Imipenem	S	S	S
	Meropenem	S	S	S
Polyphosphates	Fosfomycin	R	R	R
Glycopeptides	Vancomycin	S	S	S
Aminoglycosides	Amikacin	S	S	S
	Gentamicin	S	S	S
Tetracyclines	Tetracycline	S	S	S
Macrolides	Clarithromycin	S	S	S
	Azithromycin	S	S	S
	Erythromycin	S	S	S
Amide alcohols	Chloramphenicol	S	S	S
Quinolones	Ciprofloxacin	S	S	S
Sulfonamides	Sulfamethoxazole	S	S	S
Nitrofurans	Nitrofurantoin	R	R	R
Rifamycins	Rifampicin	S	S	S

R, resistant; S, sensitive.

### 3.5. Pathogenicity test

**Figure 2:**
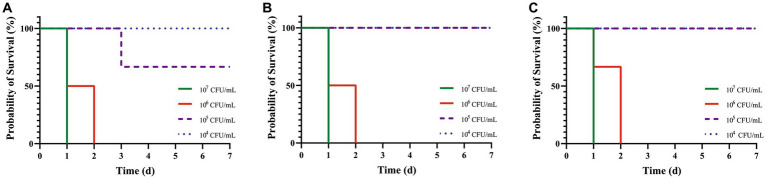
Survival curves of mice challenged with *C. pseudotuberculosis* strains at different concentrations. **(A)** G1 strain. **(B)** S2 strain. **(C)** BA3 strain.

After artificial infection with *C. pseudotuberculosis* for 5–6 h, the mice began to develop abnormal symptoms, including disheveled coat, depression, accelerated respiration, slow reaction, slow movement, and inappetence. Survival curves were plotted according to the time of death in each group (Figure 2). The LD_50_ values for mice infected with strains G1, S2 and BA3 were 1.66 × 10^5^ CFU, 3.78 × 10^5^ CFU and 3.78 × 10^5^ CFU, respectively (*p* > 0.05). The LD_50_ of strain G1 was less than that of the strains S2 and BA3, but the difference is not significant (p > 0.05).

**Figure 3:**
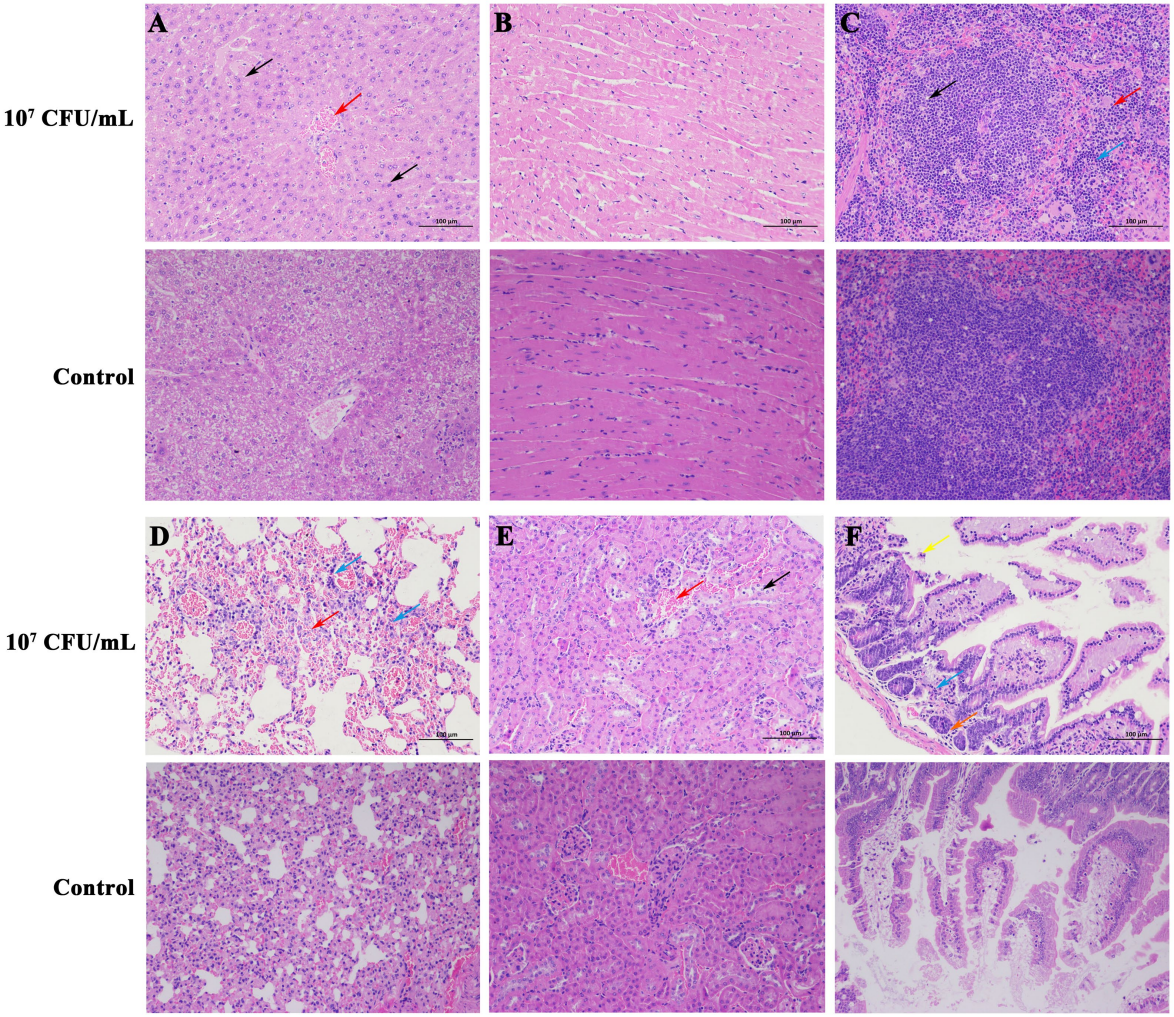
Histopathologic changes in mice killed by *C. pseudotuberculosis* (200×). **(A)** Liver. **(B)** Heart. **(C)** Spleen. **(D)** Lung. **(E)** Kidney. **(F)** Intestine.

Necropsy of dead mice (10^7^ CFU/mL group) showed dilatation of the gastrointestinal tract with significant fluid accumulation, hemorrhagic spots in the liver, and a congested and enlarged spleen. The dead mice did not show typical CLA or suppuration symptoms, which may be explained by acute death of mice post-injection and the fact that the infected organs lacked the time to develop the organ lesions. Sterile collection was performed on the liver, spleen, and kidneys of the dead mice, and after streaking and incubation, *C. pseudotuberculosis* was isolated from all organs. The pathological sections revealed that there was pronounced central vein congestion (red arrow) and diffuse hepatic cell lipid degeneration (black arrow) in the liver tissue (Figure 3A). Heart tissue (Figure 3B) showed no apparent abnormalities. Spleen tissue (Figure 3C) showed irregularities in the white pulp, along with numerous small vacuoles (black arrow) and a large area of extramedullary hematopoietic foci in the red pulp (red arrow), with a significant amount of neutrophil infiltration (blue arrow). Lung tissue (Figure 3D) exhibited extensive alveolar interstitial congestion and expansion (red arrow), with only a few lymphocyte infiltrates observed around blood vessels (blue arrow). Kidney tissue (Figure 3E) displayed a small number of epithelial cells that underwent cellular hydropic degeneration and cytoplasmic relaxation (black arrow), and a large portion of renal tubular interstitial congestion and expansion (red arrow). Intestine tissue (Figure 3F) showed a considerable amount of villous epithelial cells that experienced necrosis and shedding (yellow arrow), and there was also necrosis or atrophy of the basal intestinal glands in the lamina propria, resulting in a reduced structure (orange arrow), together with a small amount of lymphocyte infiltration (blue arrow).

**Figure 4:**
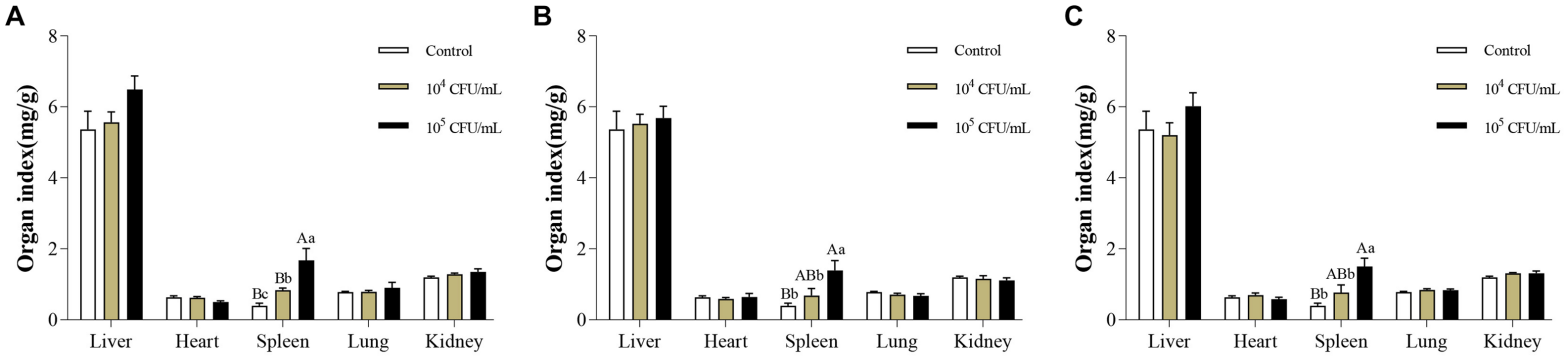
Changes of organ index in mice infected with different concentrations of *C. pseudotuberculosis*. **(A)** G1 strain. **(B)** S2 strain. **(C)** BA3 strain. Significant differences were observed among the different letter marks. Uppercase letters indicate *p* < 0.01, while lowercase letters indicate *p* < 0.05. The absence of any letter mark, or the presence of the same letter mark, indicates no significant difference (*p* > 0.05).

14 days after intraperitoneal injection of *C. pseudotuberculosis*, enlarged spleens of the mice were observed at necropsy. Changes in organ indices were consistent with necropsy findings. After intraperitoneal injection of strain G1, the spleen organ index of mice in 10^5^ CFU/mL group was significantly higher than in the control group and 10^4^ CFU/mL group (*p* < 0.01), and 10^4^ CFU/mL group was significantly higher than the control group (*p* < 0.05). After intraperitoneal injection of S2 or BA3 strains, the spleen organ index of mice in 10^5^ CFU/mL group was significantly higher than the control group (*p* < 0.01) and 10^4^ CFU/mL group (*p* < 0.05), while there was no significant difference between 10^4^ CFU/mL group and the control group (*p* > 0.05). There was no significant difference in the organ index of other organs (liver, heart, lung and kidney) (*p* > 0.05) (Figure 4). The spleen is an important immune organ in the host, and when exposed to external infections, immune cell infiltration, hypersplenism and portal hypertension can cause splenomegaly (Wang X. et al., 2021).

### 3.6. Whole genome sequencing

Based on the consistent biochemical properties, biovars identification, and antibiotic sensitivity test results of these three *C. pseudotuberculosis* strains, and no significant difference in LD_50_ among them, only one strain was specifically chosen for whole genome sequencing and comparative genomics analysis. The strain G1 was chosen due to its lower LD_50_ compared to the other two strains.

#### 3.6.1. Genome sequencing and assembly of *Corynebacterium pseudotuberculosis* strain G1

Initially, a total of 1,314 Mb of raw data were produced from the DNBSEQ sequencing platform, from 8,764,210 reads (Supplementary Table S2). After single-molecule nanopore DNA sequencing and data clean-up, 2,331,063,777 bp of sequence data were obtained, with reads mean length of 6,908 bp (Supplementary Table S3). The final assembly was composed of a chromosomal scaffold of 2,379,166 bp (52.06% G + C) (Supplementary Table S4; Supplementary Figure S5) without any plasmid. The genome has been uploaded to the NCBI (National Center for Biotechnology Information) database with accession number CP121342.

#### 3.6.2. Gene prediction and annotation of *Corynebacterium pseudotuberculosis* strain G1

A total of 49 tRNA-encoding genes and 4 rRNA operons (four 5S rRNAs, four 16S rRNAs and four 23S rRNAs) were identified in the chromosome (Supplementary Table S5). The results of tandem repeat prediction are presented in Supplementary Table S6. Phages were not predicted in this study. Three different CRISPR clusters were detected in the chromosome of *C. pseudotuberculosis* strain G1, and details are shown in Supplementary Table S7.

After comparing the ORFs to the ARDB database, we annotated that the strain G1 contains five ARGs, including *rpoB*, *rbpA*, EF-Tu mutant, *thyA*, and *murA* (identity >60%), and is predicted to be resistant to rifamycin, elfamycin, salicylic acid, and fosfomycin. In addition, we compared the strain G1 with the VFDB database and annotated 43 genes related to virulence factors, including adherence (such as *spaC* and *spaI*), exotoxin (*pld*), immune modulation (*rfbB* and *ndk*), nutritional/metabolic factor (such as *fagA*-*D* and *ciuA*-*E*), and stress survival (such as *sigE*, *sigH*, and *sigA/rpoV*). Specific virulence genes are shown in Supplementary Table S8 (identity >60%). Furthermore, we aligned the strain G1 with the I37 strain (accession number: CP017384) to annotate the virulence factor-related genes *oppA*, *oppB*, *oppC*, *oppD*, and *oppF* (identity >98%).

After comparing the predicted gene set with the COG database, the homologous gene annotation was completed, and the COG functional clustering was obtained. The genome of strain G1 has been annotated with 1538 genes that have known functions and 55 genes that have unknown functions. The highest number of genes is related to metabolism (*n* = 896), followed by cellular and information. There are 98 genes predicted for general function prediction only (Supplementary Figure S6A). Through the annotation in the GO database, we can determine the potential function of a gene based on its annotation in various categories. Supplementary Figure S6B displays the statistical results of the annotation findings for the strain G1 in the three categories (cellular component, molecular function, and biological process) of the GO database. The KEGG database divides biological pathways into eight categories, with each category further subdivided and labeled with the relevant genes. This allows for easy identification of all annotated genes associated with a specific function. Supplementary Figure S6C shows the histogram resulting from the KEGG secondary classification statistics of the strain G1.

#### 3.6.3. Comparative genomics

**Figure 5:**
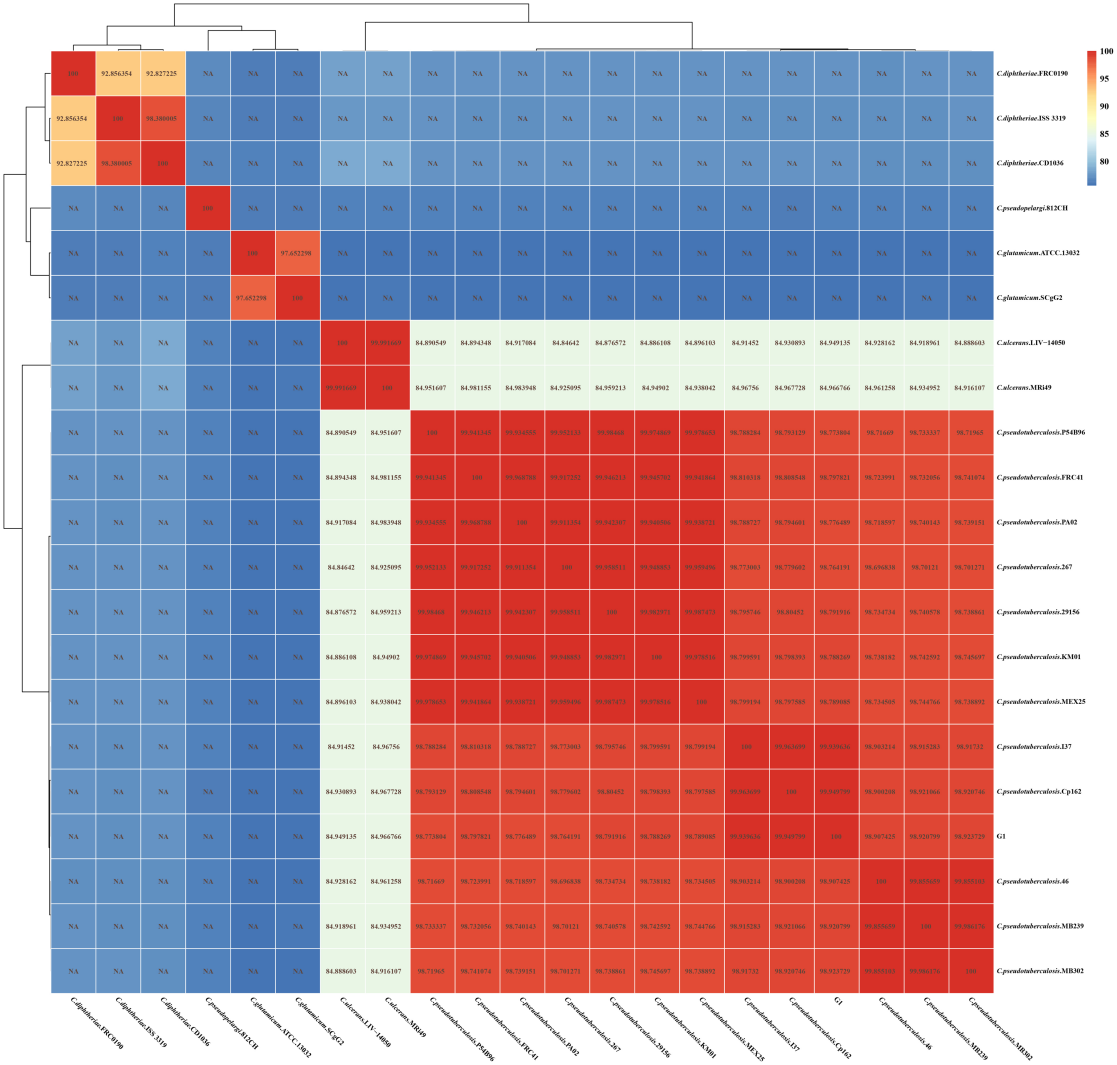
ANI heat map of 21 *Corynebacterium* strains. The horizontal and vertical axes represent the strain names. Each value in the heat map represents the ANI value.

As shown in Figure 5, strain G1 clusters with the other 12 strains of *C. pseudotuberculosis*, with intraspecies ANI values greater than 98.6. The highest ANI values (> 99.94) were observed between strain G1 and strain Cp162 (equi, camel), followed by >99.93 with strain I37 (equi, cattle). ANI values were greater than 98.9 between strains of the same biovars of *C. pseudotuberculosis*, and ranged from 98.6 to 98.9 between strains of different biovars of *C. pseudotuberculosis*. The interspecies ANI values between *C. pseudotuberculosis* and *C. ulcerans* ranged from 84.8 to 85.0. Interspecies ANI values were less than 80 for strains of *C. pseudotuberculosis* versus *C. diphtheriae*，*C. glutamicum* and *C. pseudopelargi*.

Gene family statistics for the 21 strains of *Corynebacterium* are presented in Supplementary Table S9 and Supplementary Figure S8. The analysis revealed a total of 1,080 homologous gene families, with 762 Single Copy Orthologs identified among all 20 strains of *Corynebacterium*. Notably, the strain G1 showed 8 unique paralogs and 2 unique families.

**Figure 6:**
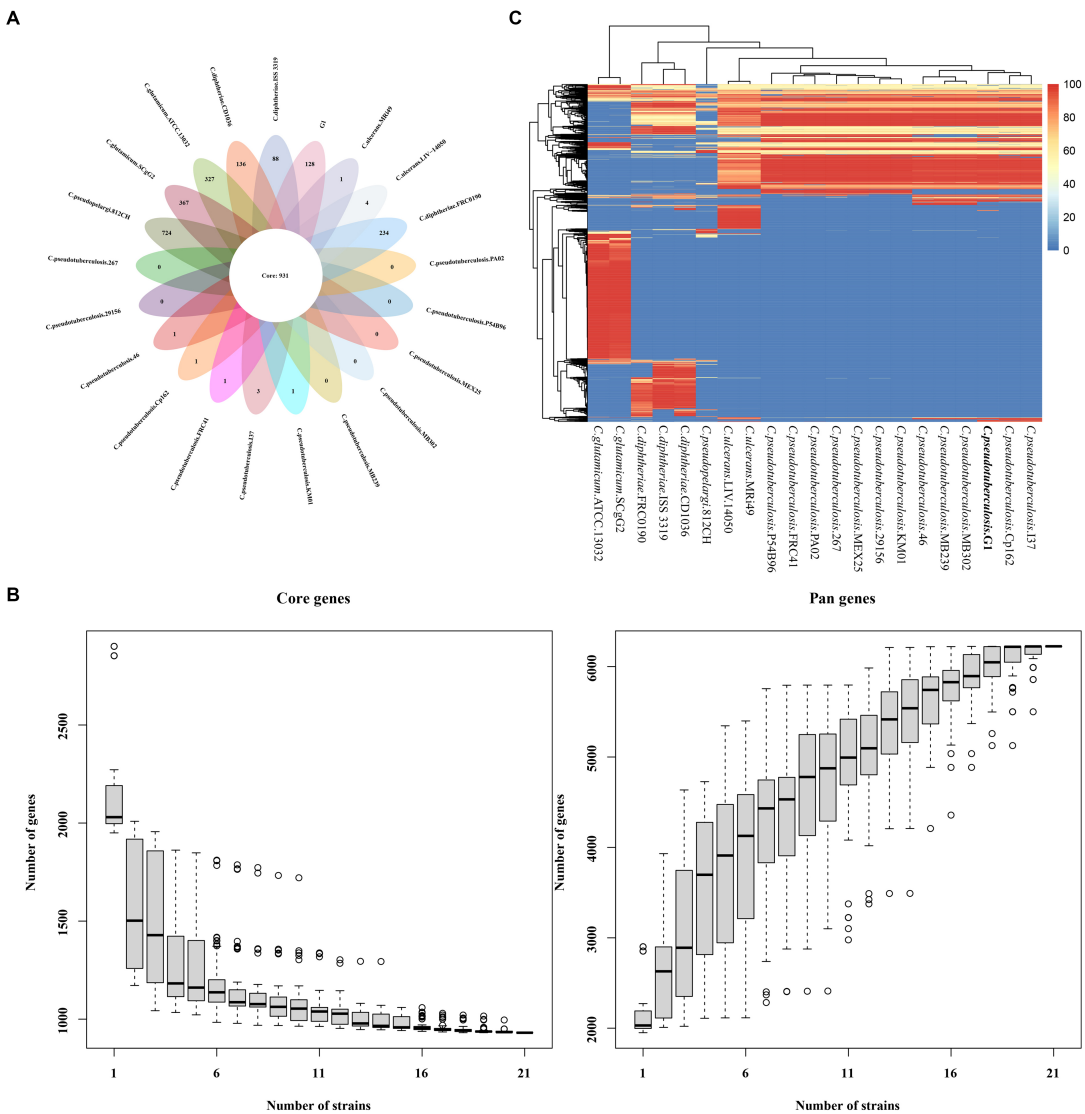
The core and pan-genome of 21 *Corynebacterium* strains. **(A)** Venn plots showing the number of core genes and strain-specific genes in 21 the *Corynebacterium* strains. **(B)** Dilution curve of the pan-genome and core genome of the 21 *Corynebacterium* strains. **(C)** Heat map displaying the dispensable genes.

The protein coding DNA sequences (CDSs) of strain G1, 12 strains of *C. pseudotuberculosis* and 8 strains of other *Corynebacterium* were clustered, and the number of pan genes was 6224, the number of core genes was 931, and the number of dispensable genes was 3277 (Figure 6A). Analyses of the core gene set and pan gene set of different numbers of strain combinations, resulted in the core gene and pan gene dilution curves (Figure 6B). According to the distribution of dispensable genes in different samples, a heat map was drawn to show the clustering among samples (Figure 6C). Different species of *Corynebacterium* were clustered separately, but different biovars of *C. pseudotuberculosis* were not clustered.

Mapped sequence library genes of the core genes, dispensable genes, and specific genes to the corresponding functions of the COG database are shown in Supplementary Figure S7. Of the 128 specific genes in *C. pseudotuberculosis* strain G1, only one gene (Cell wall/membrane/envelope biogenesis) matched the COG database. In the core and dispensable gene sequence libraries of the 21 *Corynebacterium* strains, 850 and 1934 genes, respectively, matched the COG database, with the majority of core genes (91.30%, *n* = 931) and dispensable genes (59.02%, *n* = 3277) having COG classification information. Translation, ribosomal structure and biogenesis genes had the largest proportion among the core genes (16.00%), while inorganic ion transport and metabolism genes had the highest proportion among the dispensable genes (7.93%).

**Figure 7:**
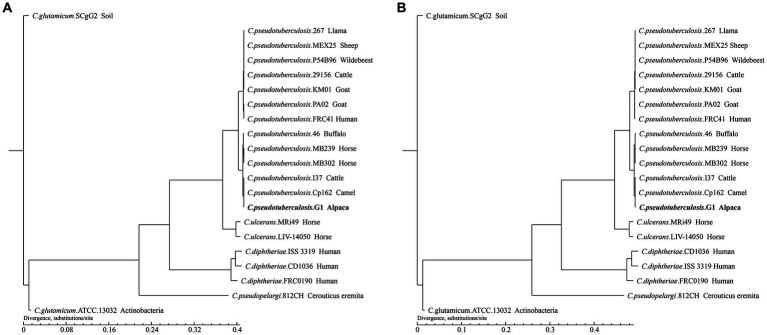
Phylogenetic tree of the 21 *Corynebacterium* strains. The tree was constructed using TreeBeST and PHYML based on **(A)** Core-pan gene. **(B)** Gene family. The numbers on the branches represent percent bootstrapping values from 1000 replicates. Each sequence is identified by its species, strain name, and host. Sequences obtained in this study are shown in bold.

To infer the phylogenetic relationships of strain G1 and 20 strains of *Corynebacterium*, we constructed phylogenetic trees based on gene family and core-pan genome (Figure 7).

The phylogenetic tree shows that different species of *Corynebacterium* clustered separately, while 13 *C. pseudotuberculosis* strains were divided into two biovars with clear demarcation, and strain G1 with *C. pseudotuberculosis* biovar equi clustered in the same clade. On the phylogenetic tree, *C. pseudotuberculosis* strain G1 was the closest relative to Cp162 and I37, and this result was consistent with the ANI analysis. The phylogenetic trees constructed based on the results of the two analyses showed the same branching structure, indicating good conservation of core-pan genes and gene families among these *Corynebacterium* strains.

**Figure 8:**
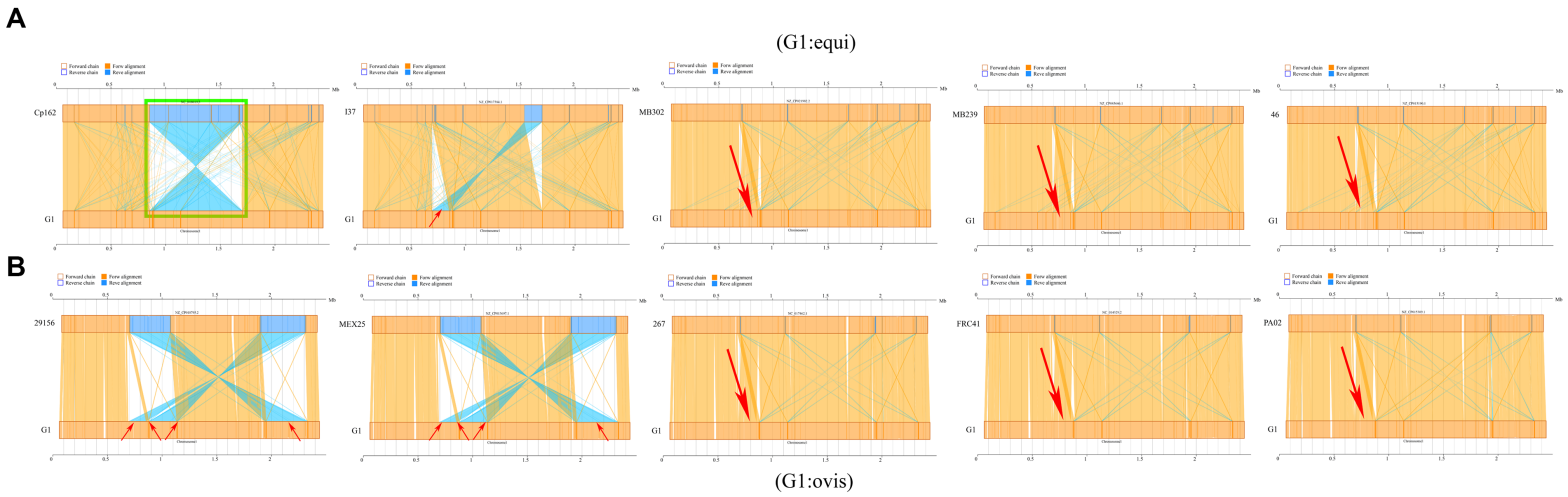
Synteny analysis. **(A)** Comparison of strain G1 with *C. pseudotuberculosis* biovar equi strains (Cp162 - camel, I37 - cattle, MB302 - horse, MB239 - horse, 46 - buffalo). **(B)** Comparison of strain G1 with *C. pseudotuberculosis* biovar ovis strains (29156 - cattle, MEX25 - sheep, 267 - llama, FRC41 - human, PA02 - goat). Green box indicates chromosome inversion, while red arrow represents chromosome translocation.

Synteny analysis was conducted between strain G1 and other *C. pseudotuberculosis* strains. The analysis revealed that strain G1 has a chromosomal inversion compared with strain Cp162 (equi, camel), a chromosomal translocation with four biovar equi (I37, MB302, MB239, and 46) and three biovar ovis (267, FRC41, and PA02) strains, and four chromosomal translocations with two biovar ovis (29,156 and MEX25) strains (Figure 8). No host or biovar-specific patterns were observed.

## 4. Discussion

CLA, caused by *C. pseudotuberculosis*, is one of the most important bacterial infectious diseases in camelids. This disease is characterized by abscesses of one or more superficial lymph nodes, and it can also infect internal organs such as the liver, lungs, and mammary glands, causing great losses to the breeding industry and attracting the attention of many scholars (Kinne, 2016). CLA has been reported in Old World Camels (Bactrian camel and dromedary camel) from several countries, such as China (Mullali et al., 2017), Russia, India (Purohit et al., 1985), Iran (Esterabadi et al., 1975), Saudi Arabia (Radwan et al., 1989), United Arab Emirates (Afzal et al., 1996), Oman, Jordan (Hawari, 2008), Kazakhstan (Samartsev, 1950), Egypt (Nashed and Mahmoud, 1987), Ethiopia (Domenech, 1980), Kenya (Wernery, 2012), Spain (Tejedor-Junco et al., 2008), as well as in Australia (Wernery et al., 2014). CLA has also been reported in New World Camels (alpaca and llama) from the USA (Lopes et al., 2012), Peru (Braga et al., 2006), Sweden, Germany (Sting et al., 2022) and Italy. Specifically, there have been reports of a llama in USA (Lopes et al., 2012) and 130 alpacas in Germany (Sting et al., 2022) being infected with biovar ovis strains. CLA by *C. pseudotuberculosis* biovar equi has not previously been reported in alpacas. Previous reports have solely documented the infection of goats with *C. pseudotuberculosis* biovar ovis strains in Sichuan Province, China (Zhang et al., 2015). While the alpacas in this study were imported from Chile and the Netherlands, it is worth noting that in Chile, cases of *C. pseudotuberculosis* biovar equi strains infection in horses have been reported (Cavalcante et al., 2015). Therefore, the pathogenic bacteria isolated in this study may have originated from the country from where the alpacas were introduced. Horses can only be infected by *C. pseudotuberculosis* biovar equi strains, while cattle and camelids can be infected by both biovars. It is crucial to be vigilant, as the outbreak of this pathogen in Chinese farms would pose a significant threat to the cattle, horse, and camelid breeding industries, resulting in economic losses such as death of farmed animals and reduced production. Therefore, effective prevention and control measures must be taken to prevent the spread and infection of this pathogen.

The genome of strain G1 is approximately 2.379 Mb, which is smaller than the genomes of biovar equi strains isolated from buffaloes (strains 31–36, 38, 39, and 48) and camel (strain 267). The GC content is 52.06%, which is lower than that of previously sequenced whole genomes of *C. pseudotuberculosis* strains (Supplementary Table S1). Three CRISPR clusters were detected on the chromosome of the strain G1, which were only detected in biovar equi strains (Parise et al., 2018). The gene set was subjected to COG functional annotation, and the categorical statistics were similar to those of six biovar equi strains (MEX1, MEX9, MEX25, MEX29, MEX30, and MEX31) isolated from equines in Mexico, except for strain MEX30 which harbors a gene related to chromatin structure and dynamics gene (Parise et al., 2018).

The widespread prevalence and significant economic impact of this disease have prompted investigations into the molecular mechanisms of virulence in this pathogen (Dorella et al., 2009). In sheep and goats, the virulence factor-related genes *pld* and *fagA*, *B* were detected in 100% of isolates, and *fagC*, *D* genes were detected in more than 95% (Aquino De Sá et al., 2013). Notably, all *fagD*-negative isolates were obtained from superficial abscesses, suggesting variations in the virulence potential of the clinical isolates (Aquino De Sá et al., 2013). A mutation in *fagB* (*C*) can reduce the pathogenicity of *C. pseudotuberculosis* in goat infections (Billington et al., 2002). To study the role of Opp in *C. pseudotuberculosis*, an OppD-deficient strain was created, and it was observed that the mutant strain had impaired growth when exposed to the toxic glutathione peptide, similar to the wild-type strain. The *ΔoppD* strain also showed a reduced ability to adhere to and infect macrophages compared to the wild-type strain, but both strains had similar potential to colonize spleens and cause injury and death to infected mice (Moraes et al., 2014).The above ten virulence genes (*pld*, *fagA*-*D*, *oppA*-*F*) were detected in the strain LY20 isolated from a goat in China (Wang R. et al., 2021). Whole genome sequencing of *C. pseudotuberculosis* strain I37 isolated from cattle (accession number CP017384, Israel) also revealed the presence of these ten virulence genes. At present, there are few reports related to *C. pseudotuberculosis* from alpaca, and the results of whole genome sequencing of *C. pseudotuberculosis* isolated from llama showed the presence of the *pld* and *fagA*-*D* (Lopes et al., 2012). In this study, we annotated 48 genes related to virulence factors in five categories (adherence, exotoxin, immune modulation, nutritional/metabolic factor, and stress survival). Among them, *pld* is the only exotoxin-related virulence gene. Other genes include σ factors (*sigE*, *sigH*, *sigA/rpoV*), iron uptake and regulation-related genes (*fagA*-*D*, *ciuA*-*E*), oligopeptide permease (*oppA*-*F*), minor pilin proteins (*spaC*, *spaI*), and superoxide dismutase (*sodA*).

The success of antimicrobial treatment to CLA is poor (Kinne, 2016). Performing resistance testing of isolates can provide guidance for the use of antibiotics. At present, there have been limited studies on antibiotic susceptibility of *C. pseudotuberculosis* in alpaca, with most research focusing on horse (Foley et al., 2004; Rhodes et al., 2015), sheep (Gallardo et al., 2019), and goat (Connor et al., 2000; Abebe and Sisay, 2015) isolates. The minimum inhibitory concentration (MIC) of the antimicrobial agent showed no significant differences between the isolates from horses and cows compared to those from sheep and goats, except in the case of amikacin (Costa et al., 1998). The MIC of equine *C. pseudotuberculosis* isolates demonstrated that several commonly used antimicrobials are effective against *C. pseudotuberculosis in vitro*. Abscess location was not associated with different MIC patterns in cultured isolates (Rhodes et al., 2015). It has been determined that 59 *C. pseudotuberculosis* isolates from sheep and goats were primarily susceptible to norfloxacin (77.97%), doxycycline HCl (72.88%), and kanamycin (72.88%), while mainly resistant to ampicillin (28.81%), clindamycin (25.42%), and doxycycline HCl (22.04%) (Abebe and Sisay, 2015). Strain XH02 (Boer goat, China) exhibited high sensitivity to 13 antibacterial agents, including chloromycetin, tetracycline, norfloxacin, minocycline, cefoxitin, clarithromycin, roxithromycin, and ceftriaxone, but completely resistant to nitrofurantoin and furazolidone (Zhou et al., 2016). However, the susceptibility of five alpaca isolates to gentamicin, sulfonamide, oxacillin, neomycin, and ceftiofur varied (Anderson et al., 2004). In this study, 21 antibiotics in 12 categories commonly used in clinical practice were selected for antibiotic susceptibility testing. The three strains were sensitive to penicillin, cefotaxime, meropenem, erythromycin and other antibiotics, resistant to aztreonam, fosfomycin, and nitrofurantoin. Combined with the antibiotic sensitivity results and clinical manifestations of the sick alpaca (S2), meropenem with high sensitivity was selected for treatment, and the sick alpaca symptoms improved, and gradually recovered.

Horizontal gene transfer (HGT) of drug-resistance genes leads to global infections of multi-drug resistant (MDR) microorganisms in hospitals and communities, posing a serious potential hazard to public health safety (Warnes et al., 2012). PCR amplification of the ARGs in *C. pseudotuberculosis* strains obtained from goats and sheep from Egypt indicated the presence of the β-lactams resistance gene (*bla*) in 40% of the isolates, and the aminoglycoside resistance gene (*aadA2*) in 42% of the isolates (Tawab et al., 2019). Upon analyzing the complete genome sequence of the *C. pseudotuberculosis* strain Cp267 isolated from llama (accession number CP003407, USA), we discovered that it carries 12 ARGs, mainly expressed as MRD protein (*marR*, *norM*, and *emrB*), lincomycin resistance protein (*lmrB*), bleomycin resistance protein and glycopeptide antibiotic resistance protein, and no β-lactam resistance gene was found (Lopes et al., 2012). In this study, the complete genome sequence of the *C. pseudotuberculosis* strain G1 isolated from alpaca was annotated with five ARGs, including rifamycin resistance genes *rpoB* and *rbpA*, elfamycin antibiotic gene EF-Tu mutant, fosfomycin antibiotic gene *murA*, and pyrazine antibiotic gene *pncA.* Our findings indicate that these ARGs exhibited a low correlation with the resistance phenotype, suggesting that detection of ARGs cannot replace routine antibiotic susceptibility testing. The expression of ARGs is influenced by various factors, including gene expression level, drug resistance mechanism, and bacterial adaptability to the environment. Therefore, the presence of a certain ARG does not necessarily imply that the bacterium will exhibit resistance to the corresponding antibiotic. Lastly, the difference in the carriage of resistance genes between the strains analyzed in this study and previous studies may be attributed to the variation in antibiotic usage in different regions to combat bacterial infectious diseases.

Several different serological tests (haemagglutination, haemagglutination inhibition, and ELISAs) have been tried for the diagnosis of CLA but, with the exception of ELISA, are rarely used on camelids (Kinne, 2016). An in-house ELISA was developed based on commercially available ELITEST, using protein A substituted anti-goat/sheep conjugate, is currently routinely used to detect CLA antibodies in dromedaries with good results (Berlin et al., 2015). Serologic testing was performed on 232 alpacas using a commercially available ELISA based on PLD as antigen and an in-lab ELISA based on whole cell antigens (WCA), and showed a substantial degree of agreement of 89.5% for both tests (Sting et al., 2022). Further comparative studies showed that the immunoblot had a sensitivity superior to both ELISAs (Sting et al., 2022). In conclusion, serological testing using ELISA combined with validation by immunoblot should be considered as critical methods to control CLA in alpaca herds.

## 5. Conclusion

To our knowledge, this is the first report of infection with *C. pseudotuberculosi* from alpaca in China, and the first whole genome sequencing of *C. pseudotuberculosis* biovar equi strain in alpaca. Comparative genomics analysis based on ANI value, gene families, core-pan genome, and synteny analysis demonstrated a high degree of genetic similarity among *C. pseudotuberculosis* strains, in contrast to other *Corynebacterium* species, with a clear delineation between strains belonging to the two biovars (ovis and equi). This study provides fundamental information for further exploration of the pathogenic mechanisms of *C. pseudotuberculosis* in alpaca and practical guidance for the prevention, diagnosis, and treatment of CLA in this species.

## Data availability statement

The original contributions presented in the study are included in the article/Supplementary material, further inquiries can be directed to the corresponding authors.

## Ethics statement

The animal study was reviewed and approved by the Sichuan Agricultural University Animal Ethical and Welfare Committee. Written informed consent was obtained from the owners for the participation of their animals in this study.

## Author contributions

WM: methodology, data analysis and visualization, and writing – original draft. SC: clinical case handling. LH: writing – original draft. JY: data analysis and visualization. WZ: investigation. ZhZ and ZiZ: resources. HL and HF: writing – review and editing. TH: project administration. GP: conceptualization and supervision. All authors have read and approved the manuscript.

## Funding

This research was supported by Sichuan Wolong National Nature Reserve Administration (510000-02-064387), and the State Forestry and Grassland Administration (2022-2222219002).

## Conflict of interest

The authors declare that the research was conducted in the absence of any commercial or financial relationships that could be construed as a potential conflict of interest.

## Publisher’s note

All claims expressed in this article are solely those of the authors and do not necessarily represent those of their affiliated organizations, or those of the publisher, the editors and the reviewers. Any product that may be evaluated in this article, or claim that may be made by its manufacturer, is not guaranteed or endorsed by the publisher.

## Footnotes

^1^ http://www.ncbi.nlm.nih.gov/BLAST

^2^ http://www.clustal.org/

^3^ https://www.broadinstitute.org/gatk/

^4^ http://www.cbcb.umd.edu/software/glimmer/

^5^ http://gtrnadb.ucsc.edu/

^6^ http://tandem.bu.edu/trf/trf.html

^7^ http://www5.esu.edu/cpsc/bioinfo/software/GIST/

^8^ http://phast.wishartlab.com/

^9^ https://crisprcas.i2bc.paris-saclay.fr//CrisprCasFinder/Index

^10^ http://treesoft.svn.sourceforge.net/viewvc/treesoft/trunk/treebest/
