# Supplementary material for: Isolation, characterization, and pathogenicity assessment of Corynebacterium pseudotuberculosis biovar equi strains from alpacas (Vicugna pacos) in China
Source: Front Microbiol. 2023 Jul 3;14:1206187. doi: 10.3389/fmicb.2023.1206187 (PMC10350510; doi:10.3389/fmicb.2023.1206187)
Supplement: Supplementary file 1 [file Data_Sheet_1.PDF]

Supplementary Material

1 Supplementary Data

Partial sequences of the 16S rRNA gene have been deposited in GenBank with accession numbers OQ980206, OP903368, and OP903369. Partial sequences of the *narG* gene have been deposited in GenBank with accession numbers OQ817706-OQ817708. The genome of strain G1 has been uploaded to the NCBI database with accession number CP121342.

2 Supplementary Figures and Tables

2.1 Supplementary Figures

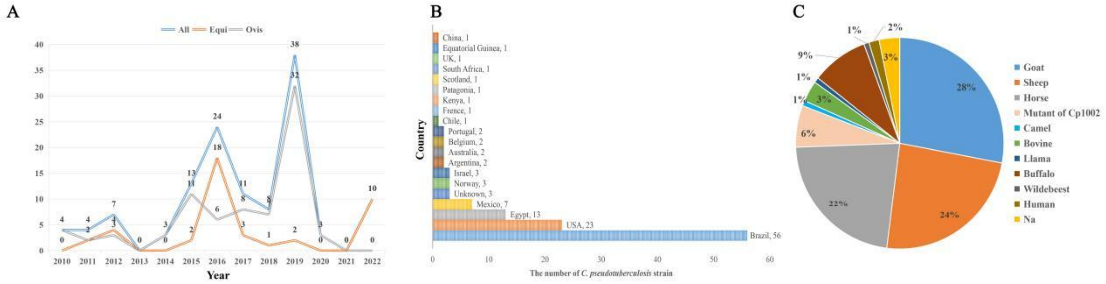

Supplementary Figure S1. Release date, geographic distribution, and hosts origin of 125 *C. pseudotuberculosis* strains with complete genome. **(A)** Release date. **(B)** Countries that have conducted whole-genome sequencing of *C. pseudotuberculosis*. **(C)** Hosts origin.

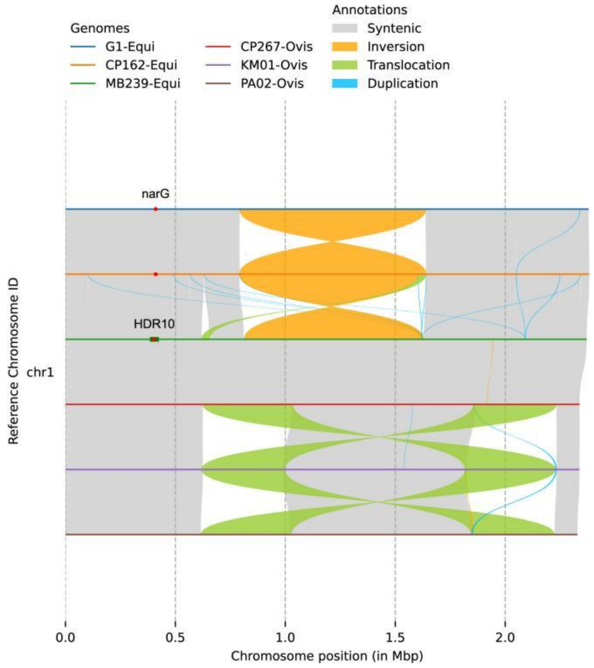

Supplementary Figure S2. High divergence regions of 6 strains of *C. pseudotuberculosis*.

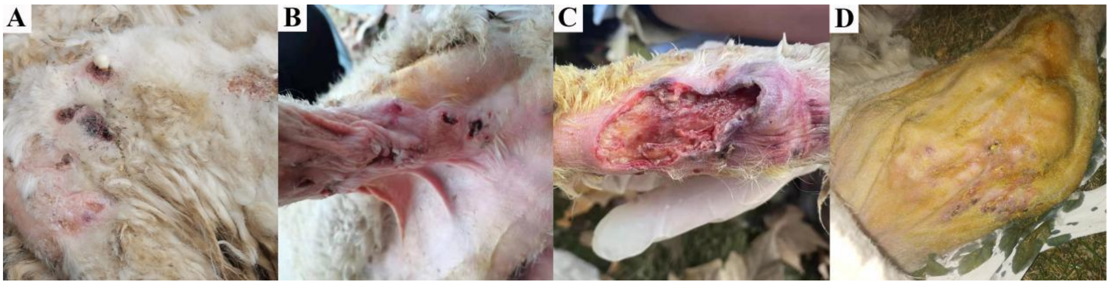

Supplementary Figure S3. Body surface examination of alpacas. **(A)** Superficial abscesses. **(B)** Abscesses that developed in a "beaded" pattern. **(C)** Skin ulceration and necrosis. **(D)** Abscesses in subcutaneous tissues.

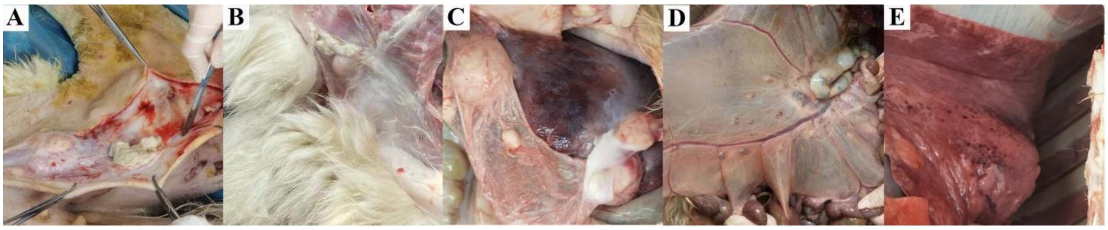

Supplementary Figure S4. Necropsy of dead alpacas. **(A)** Caseous abscess in subcutaneous tissue. **(B)** Abscesses in lymph nodes. **(C)** Liver congestion, caseous abscess around. **(D)** Suppuration in mesenteric lymph nodes. **(E)** Pulmonary hemorrhage.

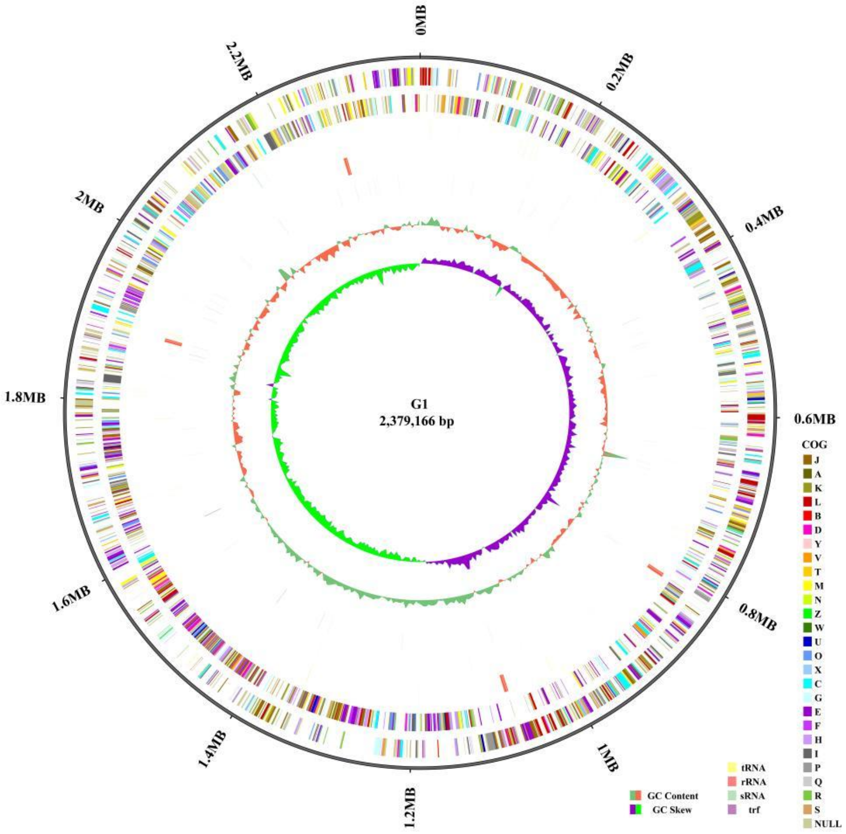

Supplementary Figure S5. Genome landscape of *C. pseudotuberculosis* strain G1.

J: Translation, ribosomal structure and biogenesis.

A: RNA processing and modification

K: Transcription

L: Replication, recombination and repair;

B: Chromatin structure and dynamics

D: Cell cycle control, cell division, chromosome partitioning

Y: Nuclear structure

V: Defense mechanisms

T: Signal transduction mechanisms

M: Cell wall/membrane/envelope biogenesis

N: Cell motility

Z: Cytoskeleton

W: Extracellular structures

U: Intracellular trafficking, secretion, and vesicular transport

O: Posttranslational modification, protein turnover, chaperones

X: Mobilome: prophages, transposons

C: Energy production and conversion

G: Carbohydrate transport and metabolism

E: Amino acid transport and metabolism

F: Nucleotide transport and metabolism

H:Coenzyme transport and metabolism

I: Lipid transport and metabolism

P: Inorganic ion transport and metabolism
Q: Secondary metabolites biosynthesis, transport and catabolism
R: General function prediction only
S: Function unknown
NULL: Unclassified

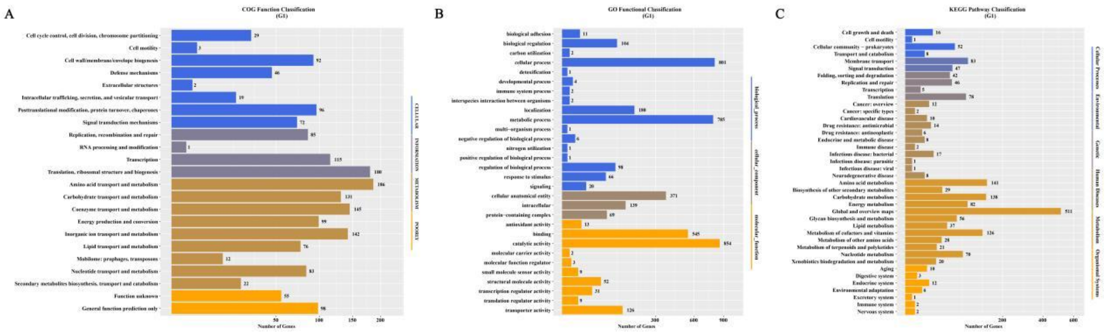

Supplementary Figure S6. General function annotation. **(A)** COG function classification. **(B)** GO functional classification. **(C)** KEGG pathway classification.

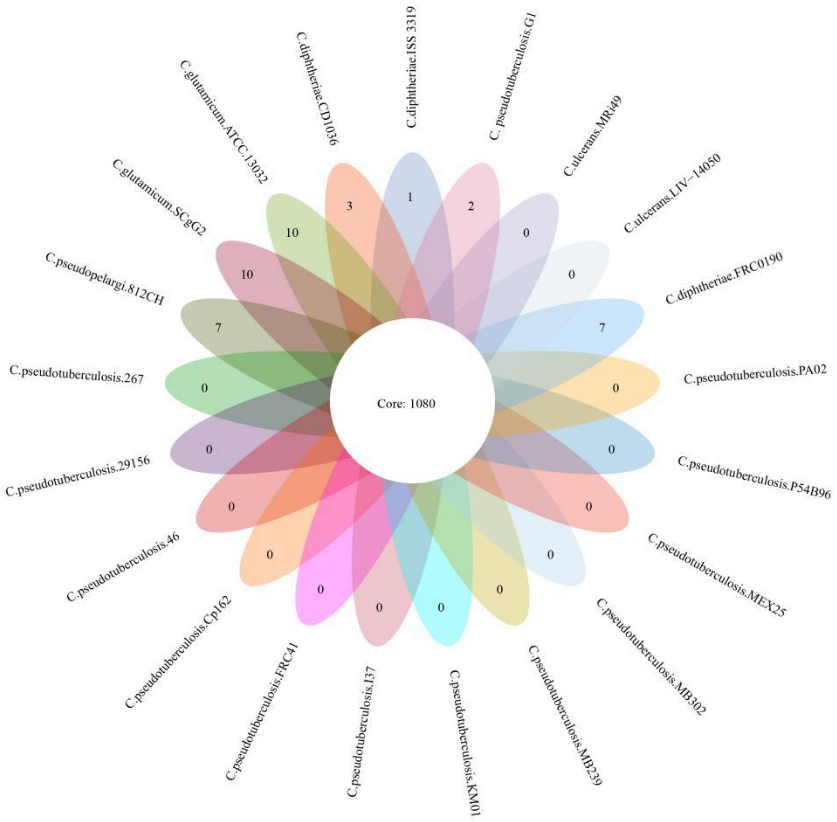

Supplementary Figure S7. Statistics of the number of homologous gene family.

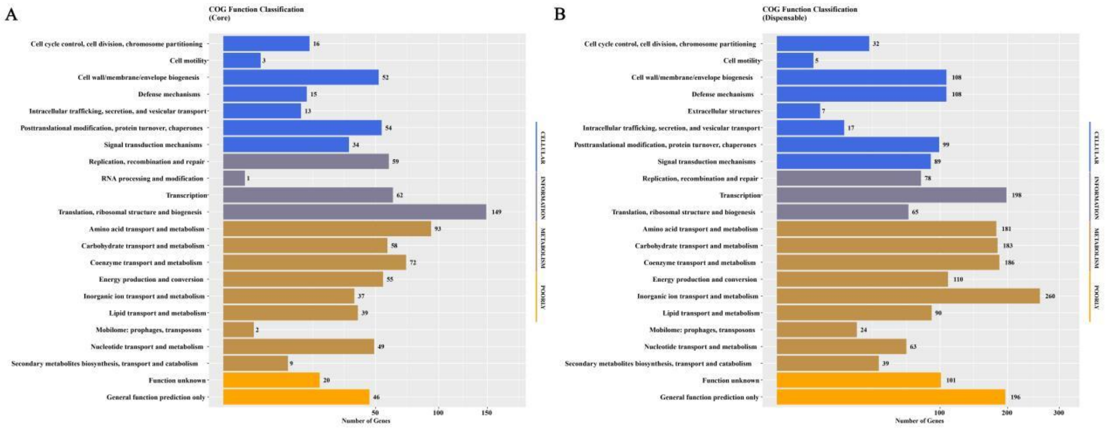

Supplementary Figure S8. COG functional annotation. **(A)** Core gene. **(B)** Dispensable gene.

2.2 Supplementary Tables

Supplementary Table S1. The genomic information of *C. pseudotuberculosis* strains that have been deposited in NCBI between 2009 and 2023.

Strain	Host	Biovar	Site of isolation	Country	Size (Mb)	GC%	CDS	Genes	other RNA	rRNA	tRNA	Pseudogene	Release Date	Replicons
**G1**	**Alpaca**	**Equi**	**Abscess**	**China**	**2.379166**	**52.06**	**2054**	**2157**	**3**	**12**	**48**	**40**	**2023-04-11**	**CP121342.1**
MB271	Horse	Equi	Kidney	USA	2.37094	52.1	2048	2156	3	12	50	0	2022-12-05	CP085645.1
**MB239**	**Horse**	**Equi**	**Liver abscess**	**USA**	**2.37058**	**52.1**	**2049**	**2153**	**3**	**12**	**50**	**0**	**2022-12-05**	**CP085646.1**
MB238	Horse	Equi	Abdominal fluid	USA	2.37058	52.1	2051	2155	3	12	50	0	2022-12-05	CP085647.1
MB325	Horse	Equi	Ulcerative lymphangitis	USA	2.37058	52.1	2050	2154	3	12	50	0	2022-12-05	CP085643.1
MB205	Horse	Equi	Triceps abscess	USA	2.36953	52.1	2048	2153	3	13	50	0	2022-12-05	CP085650.1
MB292	Horse	Equi	Multiple abscesses of spleen, kidney, liver and pector mucles	USA	2.36918	52.1	2049	2153	3	12	50	0	2022-12-05	CP085644.1
MB216	Horse	Equi	Ventral midline abscess	USA	2.36917	52.1	2047	2151	3	12	50	0	2022-12-05	CP085649.1
MB235	Horse	Equi	Abscess	USA	2.36917	52.1	2049	2152	3	12	50	0	2022-12-05	CP085648.1
MB201	Horse	Equi	Pectoral abscesses	USA	2.36917	52.1	2048	2152	3	12	50	0	2022-12-05	CP085651.1
Cp_Eq_BR 01	Horse	Equi	Nodules	Brazil	2.36995	52.1	2052	2147	3	12	50	0	2022-07-17	CP101133.1
sigC	Mutant of Cp1002	Ovis	made at Laboratory	Brazil	2.33896	52.2	2008	2117	3	12	47	47	2020-01-27	CP047926.1
SigD	Mutant of Cp1002	Ovis	made at Laboratory	Brazil	2.33931	52.2	2010	2119	3	12	47	47	2020-01-19	CP047586.1
PAT14	Sheep	Ovis	Abscess	Argentina	2.33713	52.2	2003	2112	3	12	48	46	2020-01-19	CP047603.1
SigM	Mutant of Cp1002	Ovis	made at Laboratoty	Brazil	2.33923	52.2	2007	2117	3	12	47	48	2019-12-23	CP046955.1
206	Sheep	Ovis	Missing	Brazil	2.33803	52.2	1991	2113	3	12	48	59	2019-12-23	CP046934.1
133	Sheep	Ovis	Abscess	Brazil	2.33803	52.2	1990	2112	3	12	48	59	2019-12-21	CP046861.1
414	Goat	Ovis	Caseous abscess	Brazil	2.33802	52.2	1987	2109	3	12	48	59	2019-12-21	CP046862.1
MB16	Horse	Equi	Miss	USA	2.36938	52.1	2049	2153	3	12	52	37	2019-12-18	CP046734.1

sigB	Mutant of Cp1002	Ovis	made at Laboratory	Brazil	2.33955	52.2	2009	2118	3	12	47	47	2019-12-18	CP046732.1
SigH	Mutant of Cp1002	Ovis	made at Laboratoty	Brazil	2.33926	52.2	2009	2119	3	12	47	48	2019-12-18	CP046731.1
SigK	Mutant of Cp1002	Ovis	made at Laboratoty	Brazil	2.33483	52.2	2009	2115	3	12	47	44	2019-12-18	CP046733.1
17	Sheep	Ovis	Abscess	Brazil	2.33802	52.2	1990	2113	3	12	48	60	2019-12-17	CP046643.1
SP165	Sheep	Ovis	Parotid lymph node	Brazil	2.33788	52.2	1988	2110	3	12	48	59	2019-12-17	CP046642.1
MEX2	Goat	Ovis	Left flank	Mexico	2.33755	52.2	2011	2117	3	12	48	43	2019-12-17	CP046644.1
PAT16	Sheep	Ovis	Abscess	Argentina	2.33751	52.2	2007	2114	3	12	48	44	2019-12-17	CP046641.1
E14	Goat	Ovis	retropharyngeal	Brazil	2.33816	52.2	2008	2122	3	12	48	51	2019-09-17	CP035789.1
E13	Goat	Ovis	Inguinal lymph nodes	Brazil	2.33782	52.2	2007	2121	3	12	48	51	2019-09-17	CP035745.1
E16	Goat	Ovis	Pre-scapular	Brazil	2.33781	52.2	2012	2126	3	12	48	51	2019-09-17	CP036149.1
OVICCA32	Goat	Ovis	Lymph node	Brazil	2.33816	52.2	2007	2122	3	12	48	52	2019-07-15	CP035642.1
E9	Goat	Ovis	Mammary	Brazil	2.33815	52.2	2010	2124	3	12	48	51	2019-07-15	CP035714.1
OVIZ01	Sheep	Ovis	Lymph node	Brazil	2.33786	52.2	2008	2123	3	12	48	52	2019-07-15	CP035678.1
OVIOS02	Sheep	Ovis	Lymph node	Brazil	2.33759	52.2	2023	2129	3	12	48	43	2019-07-15	CP035679.1
CAPGE03	Goat	Ovis	Lymph node	Brazil	2.33756	52.2	2021	2125	3	12	48	41	2019-07-15	CP035641.1
OVID04	Sheep	Ovis	Lymph node	Brazil	2.33754	52.2	2008	2123	3	12	48	52	2019-07-15	CP035640.1
Cap5W	Goat	Ovis	Mammary gland	Brazil	2.33758	52.2	2016	2118	3	12	48	39	2019-05-14	CP039866.1
Cap4W	Goat	Ovis	Parotid	Brazil	2.33757	52.2	2010	2114	3	12	48	41	2019-05-14	CP039867.1
NCTC4656	Horse	Equi	Missing	Unknown	2.36943	52.1	2049	2146	3	12	48	34	2019-05-11	LR590478.1
NCTC4681	Sheep	Ovis	Missing	Unknown	2.3378	52.2	2000	2111	3	12	48	48	2019-05-11	LR590479.1
OVI1FL	Sheep	Ovis	Pre-capsular lymph node	Brazil	2.33755	52.2	1990	2111	3	12	48	58	2019-04-30	CP039633.1
Cap1C	Goat	Ovis	Parotid	Brazil	2.33752	52.2	1990	2112	3	12	48	59	2019-04-11	CP038431.1
E7	Goat	Ovis	Pre-scapular	Brazil	2.33791	52.2	1989	2111	3	12	48	59	2019-03-15	CP036535.1
38MAT	NA	NA	Pre-capsular lymph node	Brazil	2.33813	52.2	1987	2109	3	12	48	59	2019-03-07	CP036457.1
04MAT	NA	NA	Pre-capsular lymph node	Brazil	2.33758	52.2	2006	2115	3	12	48	46	2019-03-07	CP036469.1
Cap1R	Goat	Ovis	Large intestine	Brazil	2.33813	52.2	1988	2110	3	12	48	59	2019-03-04	CP036258.1

Cap8W	Goat	Ovis	Mammary gland	Brazil	2.33758	52.2	2007	2115	3	12	48	45	2019-03-04	CP036257.1
99MAT	NA	NA	Pre-capsular lymph node	Brazil	2.33758	52.2	2010	2117	3	12	48	44	2019-02-25	CP036169.1
CAPNAT1	Goat	Ovis	Inguinal lymph node	Brazil	2.3376	52.2	2006	2113	3	12	48	44	2019-02-13	CP035716.1
CR07	Goat	Ovis	Retropharyngeal lymph node	Brazil	2.33759	52.2	2009	2117	3	12	48	45	2019-02-13	CP035715.1
87MAT	NA	NA	Pre-capsular lymph node	Brazil	2.33758	52.2	2007	2113	3	12	48	43	2019-02-13	CP035719.1
CAPMI05	Goat	Ovis	Miss	Brazil	2.33758	52.2	2013	2119	3	12	48	43	2019-02-13	CP035718.1
CAPMI03	Goat	Ovis	Mammary gland lymph node	Brazil	2.33753	52.2	2005	2112	3	12	48	44	2019-02-13	CP035717.1
OVIAF1	Sheep	Ovis	Pre-capsular lymph node	Brazil	2.33757	52.2	2009	2115	3	12	48	43	2018-12-19	CP034410.1
Cap1W	Goat	Ovis	Missing	Brazil	2.3375	52.2	2009	2114	3	12	48	42	2018-12-19	CP034411.1
OVI03	Sheep	Ovis	Animal lymphoma parotid	Brazil	2.33754	52.2	2010	2119	3	12	48	46	2018-02-06	CP026524.1
OVI2C	Sheep	Ovis	Animal lymphoma pre-scapular	Brazil	2.33758	52.2	2009	2114	3	12	48	42	2018-02-05	CP026374.1
CAPJ4	Goat	Ovis	Animal lymphoma retrofaringeo	Brazil	2.33755	52.2	2011	2117	3	12	48	43	2018-02-05	CP026499.1
CAP3W	Goat	Ovis	Animal lymphoma scapular	Brazil	2.3375	52.2	2013	2115	3	12	48	39	2018-02-05	CP026500.1
MB295	Horse	Equi	Arthritis in hock	USA	2.36925	52.1	2057	2147	3	12	48	27	2018-01-30	CP026501.1
**KM01**	**Goat**	**Ovis**	**Abscess**	**China**	**2.33767**	**52.2**	**2007**	**2113**	**3**	**12**	**48**	**43**	**2018-01-09**	**CP024995.1**
PA08	Sheep	Ovis	lymph node	Brazil	2.33776	52.2	1994	2105	3	12	48	48	2017-11-21	CP024602.1
PA07	Sheep	Ovis	Caseous secretion	Brazil	2.33735	52.2	1988	2103	3	12	48	52	2017-10-30	CP024457.1
MB154	Horse	Equi	Pectoral abscess	USA	2.37121	52.1	2004	2154	3	12	47	88	2017-10-27	CP024442.1
MB278	Horse	Equi	Renal abscess	USA	2.3699	52.1	2048	2149	3	12	47	39	2017-09-18	CP023395.1
**MB302**	**Horse**	**Equi**	**Liver abscess**	**USA**	**2.36917**	**52.1**	**2047**	**2149**	**3**	**12**	**50**	**37**	**2017-06-20**	**CP021982.2**
ATCC 19410	Sheep	Ovis	Abscess	Unknown	2.33776	52.2	2018	2121	3	12	48	40	2017-05-22	CP021251.1
SigmaE	Mutant of Cp1002	Ovis	made at Laboratoty	Brazil	2.33926	52.2	2010	2121	3	12	47	49	2017-03-27	CP020356.1
phoP	Goat	Ovis	Abscess	Brazil	2.3393	52.2	2009	2116	3	12	47	45	2017-03-02	CP019768.1
MIC6	Sheep	Ovis	Abscess	Brazil	2.33715	52.2	2012	2113	3	12	48	38	2017-03-02	CP019769.1
PA04	Sheep	Ovis	Mandibular lymph node	Brazil	2.33809	52.2	2002	2122	3	12	48	57	2017-02-07	CP019587.1
MEX1	Goat	Ovis	Retropharyngeal abscess	Mexico	2.33709	52.2	2025	2131	3	12	48	43	2017-01-30	CP017711.1
MEX30	Horse	Equi	Lymph node, chronical abscesses	Mexico	2.36814	52.1	2035	2166	3	12	50	66	2016-12-27	CP017291.1

MEX31	Horse	Equi	Lymph node, chronical abscesses	Mexico	2.36788	52.1	2066	2156	3	12	47	28	2016-12-27	CP017292.1
**I37**	**Bovine**	**Equi**	**Mammary tissue**	**Israel**	**2.37028**	**52.1**	**2030**	**2144**	**3**	**12**	**48**	**51**	**2016-12-05**	**CP017384.1**
39	Buffalo	Equi	Miss	Egypt	2.40358	52.1	2103	2203	3	12	48	37	2016-11-07	CP015188.1
33	Buffalo	Equi	Miss	Egypt	2.40355	52.1	2107	2208	3	12	51	35	2016-11-07	CP015184.1
32	Buffalo	Equi	Miss	Egypt	2.40353	52.1	2106	2206	3	12	51	34	2016-11-07	CP015183.1
38	Buffalo	Equi	Miss	Egypt	2.40352	52.1	2101	2203	3	12	48	39	2016-11-07	CP015187.1
35	Buffalo	Equi	Miss	Egypt	2.4035	52.1	2104	2207	3	12	51	37	2016-11-07	CP015185.1
34	Buffalo	Equi	Miss	Egypt	2.40345	52.1	2107	2207	3	12	48	37	2016-11-07	CP015192.1
36	Buffalo	Equi	Miss	Egypt	2.40341	52.1	2106	2205	3	12	48	36	2016-11-07	CP015186.1
48	Buffalo	Equi	Miss	Egypt	2.4033	52.1	2108	2205	3	12	48	34	2016-11-07	CP015191.1
**46**	**Buffalo**	**Equi**	**Miss**	**Egypt**	**2.36657**	**52.1**	**2058**	**2155**	**3**	**12**	**47**	**35**	**2016-11-07**	**CP015190.1**
43	Buffalo	Equi	Miss	Egypt	2.36508	52.1	2059	2156	3	11	50	33	2016-11-07	CP015189.1
MEX29	Sheep	Ovis	Miss	Mexico	2.33787	52.2	2032	2124	3	12	48	29	2016-11-03	CP016826.1
MB20	Horse	Equi	Abscess	USA	2.3709	52.1	1880	2141	3	12	51	195	2016-08-11	CP016829.1
Cp13	Goat	Ovis	Caseous lymphadenitis	Brazil	2.34224	52.2	2017	2120	3	12	48	40	2016-07-08	CP014998.1
T1	Goat	Ovis	Miss	Brazil	2.33758	52.2	2019	2125	3	12	48	43	2016-07-08	CP015100.2
**PA02**	**Goat**	**Ovis**	**Abscess**	**Brazil**	**2.32843**	**52.2**	**2017**	**2107**	**3**	**12**	**44**	**31**	**2016-06-16**	**CP015309.1**
MEX9	Goat	Ovis	Miss	Mexico	2.33758	52.2	2027	2126	3	12	48	36	2016-05-27	CP014543.1
E55	Sheep	Ovis	Miss	Egypt	2.33538	52.2	2002	2114	3	12	48	49	2016-05-27	CP014341.1
MB66	Horse	Equi	Lymph node	USA	2.3722	52.1	1999	2169	3	12	50	105	2016-03-03	CP013263.1
MB14	Horse	Equi	Lymph node	USA	2.37076	52.1	2004	2173	3	12	50	104	2016-03-03	CP013261.1
MB11	Horse	Equi	Lymph node	USA	2.37043	52.1	2063	2167	3	12	52	37	2016-03-03	CP013260.2
MB30	Horse	Equi	Lymph node	USA	2.3681	52.1	2049	2164	3	12	47	53	2016-03-03	CP013262.2
**MEX25**	**Sheep**	**Ovis**	**Miss**	**Mexico**	**2.33753**	**52.2**	**2024**	**2125**	**3**	**12**	**48**	**38**	**2015-12-23**	**CP013697.1**
PO222/4-1	Goat	Ovis	Abscess	Portugal	2.33751	52.2	2021	2123	3	12	48	39	2015-12-23	CP013698.1
E56	Sheep	Ovis	Miss	Egypt	2.33577	52.2	2004	2113	3	12	48	46	2015-12-23	CP013699.1
PA01	Sheep	Ovis	Lymph node	Brazil	2.33792	52.2	2027	2118	3	12	48	28	2015-12-03	CP013327.1
N1	Sheep	Ovis	Lung	Equatorial Guinea	2.33785	52.2	2024	2124	3	12	48	37	2015-11-23	CP013146.1

1002B	mutant of Cp1002	Ovis	Caseous abscess	Brazil	2.33511	52.2	2022	2124	3	12	47	40	2015-11-05	CP012837.1
PO269-5	Goat	Ovis	Abscess	Portugal	2.33712	52.2	2019	2130	3	12	48	48	2015-09-24	CP012695.1
E19	Horse	Equi	Miss	Chile	2.36796	52.1	2037	2142	3	12	47	43	2015-07-24	CP012136.1
262	Bovine	Equi	Miss	Belgium	2.36112	52.1	2020	2119	3	12	47	37	2015-07-07	CP012022.2
**29156**	**Bovine**	**ovis**	**Miss**	**Israel**	**2.33799**	**52.2**	**2010**	**2116**	**3**	**12**	**48**	**43**	**2015-06-03**	**CP010795.2**
12C	Sheep	Ovis	Abscess	Brazil	2.33745	52.2	1997	2113	3	12	48	53	2015-06-02	CP011474.1
226	Goat	Ovis	Abscess	USA	2.33782	52.2	1962	2114	3	12	48	89	2015-04-16	CP010889.1
VD57	Goat	Ovis	Abscess	Brazil	2.33718	52.2	2009	2114	3	12	48	42	2015-01-06	CP009927.1
Ft_2193/67	Goat	Ovis	Pus	Norway	2.3383	52.2	2001	2114	3	12	48	50	2014-07-17	CP008924.1
CS_10	Human	Ovis	Abscess	Norway	2.33814	52.2	1992	2111	3	12	48	56	2014-07-17	CP008923.1
48252	Human	Ovis	Miss	Norway	2.33814	52.2	1996	2113	3	12	48	54	2014-07-17	CP008922.1
**Cp162**	**camel**	**Equi**	**Abscess**	**UK**	**2.3821**	**52.1**	**2037**	**2154**	**3**	**12**	**48**	**54**	**2012-06-11**	**CP003652.3**
258	Horse	Equi	Missing	Belgium	2.36888	52.1	2054	2152	3	12	50	33	2012-05-22	CP003540.3
31	Buffalo	Equi	Miss	Egypt	2.40492	52.1	2087	2194	3	12	51	41	2012-04-18	CP003421.4
**267**	**llama**	**Ovis**	**Submandibular abscess**	**USA**	**2.33763**	**52.2**	**2016**	**2106**	**3**	**12**	**48**	**27**	**2012-04-10**	**CP003407.1**
**P54B96**	**wildebeest**	**Ovis**	**Liver, lung, mediastinal, lymph node**	**South Africa**	**2.33766**	**52.2**	**1966**	**2114**	**3**	**12**	**48**	**85**	**2012-03-23**	**CP003385.1**
316	Horse	Equi	Subcutaneous abscess	USA	2.36885	52.1	2047	2150	3	12	50	38	2012-02-22	CP003077.2
3/99-5	Sheep	Ovis	Abscess	Scotland	2.33794	52.2	2003	2116	3	12	48	50	2012-01-10	CP003152.1
1/06-A	Horse	Equi	Abscess	USA	2.27912	52.2	1963	2127	0	12	49	103	2011-11-03	CP003082.1
CIP 52.97	Horse	Equi	Missing	Kenya	2.36913	52.1	2055	2152	3	12	50	32	2011-10-06	CP003061.3
42/02-A	Human	Ovis	Abscess	Australia	2.33761	52.2	2003	2116	3	12	48	50	2011-10-03	CP003062.1
PAT10	Sheep	Ovis	Abscess	Patagonia	2.33532	52.2	1998	2125	3	12	47	65	2011-07-27	CP002924.1
I19	Bovine	Ovis	Miss	Israel	2.33735	52.2	2004	2111	3	12	48	44	2010-10-12	CP002251.3
1002	Goat	Ovis	Abscess	Brazil	2.33483	52.2	2005	2111	3	12	47	44	2010-08-06	CP001809.3
C231	Sheep	Ovis	Abscess	Australia	2.33725	52.2	2000	2111	3	12	48	48	2010-08-05	CP001829.2
**FRC41**	**Human**	**Ovis**	**Inguinal lymph node**	**Frence**	**2.33724**	**52.2**	**2003**	**2113**	**3**	**12**	**48**	**47**	**2010-07-16**	**CP002097.2**

Note: The genomic information of *C. pseudotuberculosis* strains used for comparative genomics studies is highlighted in bold.

Supplementary Table S2. DNBSEQ sequencing data statistics.

Sample Name	Insert Size (bp)	Reads Length (bp)	Raw Data (Mb)	Total Reads	Filtered Reads (%)	Clean Data (Mb)
G1	350	(150:150)	1,314	8764210	5.07	1,247

Supplementary Table S3. Nanopore Reads sequencing data statistics.

Sample Name	Reads Number	Reads Total Bases (bp)	Reads Mean Length (bp)	Reads N50 (bp)	Reads N90 (bp)	Reads Max Length (bp)	Reads Min Length (bp)
G1	337432	2,331,063,777	6908	10643	2847	111974	2000

Supplementary Table S4. Assembly statistics.

Sample Name	ID Name	Sequence Type	Sequence Topology	Sequence Number	Total Length (bp)	GC Content (%)
G1	Chromosome1	Chromosome	circular	1	2,379,166	52.06
	All	All	-	1	2,379,166	52.06

Supplementary Table S5. Non-coding RNA statistics.

Sample Name	Type	Copy Number	Average Length (bp)	Total Length (bp)	In Genome (%)
G1	tRNA	49	76.06	3,727	0.1567
	5s_rRNA (Denovo)	4	116	464	0.0195
	16s_rRNA (Denovo)	4	1507	6028	0.2533
	23s_rRNA (Denovo)	4	3081.75	12327	0.518
	sRNA	1	283	283	0.0119

Supplementary Table S6. Repeating sequence statistics.

Sample Name	Type	Number	Repeat Size (bp)	Total Length (bp)	In Genome (%)
G1	TRF	52	11-339	2,867	0.1205
	Minisatellite DNA	43	15-60	2,071	0.0870
	Microsatellite DNA	0	0-0	0	0.0000

Supplementary Table S7. CRISPR system type and *cas* gene composition of the G1 strain.

CRISPR ID	Type	CRISPR Start	CRISPR End	Number of Spacer	Repeat consensus	*cas* gene
Chromosome1_1	I-E	26086	26175	1	GGAACTACCTCCGCATACGCGGAGAAAAG	cas2, cas1, cas3, cas6,
Chromosome1_2	I-E	30998	31069	1	TTCCTCGCGTCCTGCGGGGGTAG	cse2, cas7, cas5
Chromosome1_3	N	1848564	1848836	3	CTTTTCTCCGCGCACGCGGAGGTAGTTC	-

Note: N, no *cas* gene cluster near the CRISPR cluster; -, no *cas* gene.

Supplementary Table S8. Statistics of VFDB gene set annotation results (identity > 60%).

Classification	Gene	Product	Number
Adherence	*groEL2*	Molecular chaperone GroEL	1
	tufA	Elongation factor Tu	1
	srtA	Fimbrial associated sortase	1
	spaC	SpaA-type pili minor subunit SpaC	1
	spaI	SpaH-type pili minorsubunit SpaI	1
	srtC	Fimbrial associated sortase	1
	sapA	Hypothetical protein	1
Exotoxin	*pld*	Phospholipase D	1
immune modulation	*rfbB*	dTDP-glucose 4,6-dehydratase	1
	ndk	Probable nucleoside diphosphate kinase (NDK)	1
Nutritional/Metabolic factor	*fagA*	Iron ABC transporter permease	1
	fagB	Iron ABC transporter permease	1

	fagC	ABC transporter ATP-binding protein	1
	fagD	Iron-siderophore ABC transporter substrate-binding protein	1
	ciuA	ABC transporter substrate-binding protein	1
	ciuB	Iron ABC transporter permease	1
	ciuC	Iron ABC transporter permease	1
	ciuD	Iron ABC transporter ATP-binding protein	1
	ciuE	Siderophore biosynthesis related protein	1
	Rv0207c	Hypothetical protein	1
	fatD	Iron compound ABC transporter permease protein	1
	leuD	3-isopropylmalate dehydratase, small subunit	1
	glnA1	Glutamine synthetase	2
	purC	Probable phosphoribosylaminoimidazole-succinocarboxamide synthase PurC	1
	fhuC	Iron compound ABC transporter ATP-binding protein	1
	narH	Hypothetical protein	1
	hmuT	Iron ABC transporter substrate-binding protein	1
	hmuU	Iron ABC transporter permease	1
	hmuV	Iron ABC transporter ATP-binding protein	1
	srtB	Putative fimbrial associated sortase	1
	ureA	Urease alpha subunit UreA	1
	ureG	Urease accessory protein UreG	1
Stress survival	*sigE*	RNA polymerase sigma factor SigE	1
	sigH	ECF RNA polymerase sigma factor SigH	1
	sigA/rpoV	RNA polymerase sigma factor	2
	ahpC	Putative alkylhydroperoxidase C	1
	sodA	Probable superoxide dismutase (Mn)	1
	relA	PPGPP synthetase I	1
	regX3	Response regulator with CheY-like receiver domain and winged-helix DNA-binding domain	1
	dtxR	DtxR family transcriptional regulator	1
	mprA	two component transcriptional regulator, winged helix family	1
	phoP	RNA polymerase sigma factor	1
	hspR	Heat shock protein transcriptional repressor HspR	1

Supplementary Table S9. Gene family statistics.

Sample ID	Gene Number	Clustered Gene				UnClustered Gene	Family Number	Unique Family
		Single Copy Orthologs	Multiple Copy Orthologs	Unique Paralogs	Other Orthologs			
G1	2,215	762	665	8	641	139	1,662	2
C. *pseudotuberculosis*.267	2,030	762	662	0	605	1	1,642	0
C. pseudotuberculosis 29156	2,020	762	653	0	605	0	1,640	0
C. *pseudotuberculosis* 46	2,058	762	663	0	632	1	1,661	0
C. pseudotuberculosis Cp162	2,037	762	659	0	613	3	1,640	0
C. *pseudotuberculosis* FRC41	2,003	762	652	0	587	2	1,623	0
C. pseudotuberculosis.I37	2,041	762	661	0	612	6	1,648	0
C.pseudotuberculosis KM01	2,020	762	652	0	604	2	1,639	0
C.pseudotuberculosis MB239	2,063	762	662	0	638	1	1,665	0
C. *pseudotuberculosis*.MB302	2,062	762	663	0	637	0	1,666	0
C.pseudotuberculosis.MEX25	2,027	762	658	0	607	0	1,640	0
C. pseudotuberculosis.P54B96	1,981	762	638	0	577	4	1,616	0
C. pseudotuberculosis.PA02	2,018	762	653	0	601	2	1,637	0
C. ulcerans.LIV-14050	2,210	762	704	0	739	5	1,771	0
C. ulcerans.MRi49	2,209	762	705	0	742	0	1,773	0
C. diphtheriae.FRC0190	2,162	762	648	15	657	67	1,678	1
C. diphtheriae.ISS 3319	2,347	762	661	2	670	99	1,792	3
C. diphtheriae.CD1036	2,234	762	673	10	803	152	1,681	7
C. glutamicum.ATCC.13032	2,970	762	804	23	1,128	253	2,004	10
C. glutamicum.SCgG2	2,989	762	847	22	1,178	180	2,022	10
C. pseudopelargi.812CH	2,144	762	653	16	485	228	1,538	7
